# Protein Composition and Associated Material Properties of Cobweb Spiders’ Gumfoot Glue Droplets

**DOI:** 10.1093/icb/icab086

**Published:** 2021-05-18

**Authors:** Nadia A Ayoub, Kyle Friend, Thomas Clarke, Richard Baker, Sandra M Correa-Garhwal, Andrew Crean, Enkhbileg Dendev, Delaney Foster, Lorden Hoff, Sean D Kelly, Wade Patterson, Cheryl Y Hayashi, Brent D Opell

**Affiliations:** 1 Department of Biology, Washington and Lee University, Lexington, VA 24450, USA; 2 Department of Chemistry and Biochemistry, Washington and Lee University, Lexington, VA 24450, USA; 3 Sackler Institute for Comparative Genomics, American Museum of Natural History, New York, NY 10024, USA; 4 Department of Biological Sciences, Virginia Tech, Blacksburg, VA 24060, USA; 5 Joint Department of Evolutionary Biology, San Diego State University and University of California Riverside, San Diego, CA 92182, USA

## Abstract

The origin of aggregate silk glands and their production of wet adhesive silks is considered a key innovation of the Araneoidea, a superfamily of spiders that build orb-webs and cobwebs. Orb-web weavers place aggregate glue on an extensible capture spiral, whereas cobweb weavers add it to the ends of strong, stiff fibers, called gumfoot lines. Here we describe the material behavior and quantitative proteomics of the aggregate glues of two cobweb weaving species, the western black widow, *Latrodectus hesperus*, and the common house spider, *Parasteatoda tepidariorum*. For each species, respectively, we identified 48 and 33 proteins that were significantly more abundant in the portion of the gumfoot line with glue than in its fibers. These proteins were more highly glycosylated and phosphorylated than proteins found in silk fibers without glue, which likely explains aggregate glue stickiness. Most glue-enriched proteins were of anterior aggregate gland origin, supporting the hypothesis that cobweb weavers’ posterior aggregate glue is specialized for another function. We found that cobweb weaver glue droplets are stiffer and tougher than the adhesive of most orb-web weaving species. Attributes of gumfoot glue protein composition that likely contribute to this stiffness include the presence of multiple protein families with conserved cysteine residues, a bimodal distribution of isoelectric points, and families with conserved functions in protein aggregation, all of which should contribute to cohesive protein–protein interactions. House spider aggregate droplets were more responsive to humidity changes than black widow droplets, which could be mediated by differences in protein sequence, post-translational modifications, the non-protein components of the glue droplets, and/or the larger amount of aqueous material that surrounds the adhesive cores of their glue droplets.

## Introduction

The success of 4673 species of orb-web weaving spiders and of 3524 species of cobweb weaving spiders has been attributed to the presence of glue in their webs (Bond and Opell 1998; World Spider Catalog 2021). These pliable adhesives exhibit properties of soft matter, which is characterized by “complex emergent behavior, such as spontaneous pattern formation, self-assembly, and a large response to small external stimuli” (van der Gucht 2018). Cobwebs spun by members of the family Theridiidae and Nesticidae appear less highly organized than orb-webs. However, as cobweb spiders descended from an orb-web weaving ancestor, their webs and their adhesives, can be considered more specialized (Blackledge et al. 2009; Coddington et al. 2019; Kallal et al. 2021). A notable feature of theridiid cobwebs is the placement of adhesive on only short regions at the lower ends of a few threads named gumfoot lines (Argintean et al. 2006; Eberhard et al. 2008). It has been observed that the biomechanical properties and humidity responsiveness of gumfoot adhesive of one species differ from those of orb-web adhesives (Sahni et al. 2011).

**Fig. 1:**
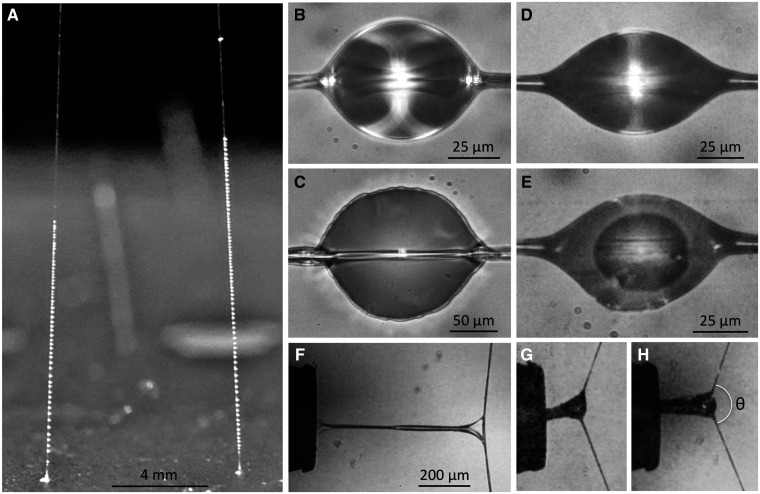
Gumfoot lines and droplets. (**A**) *Latrodectus hesperus* gumfoot lines. (**B**) Suspended *L. hesperus* glue droplet, whose convex surface magnifies its two supporting major ampullate fibers. (**C**) The same *L. hesperus* droplet flattened to reveal its adhesive core, which occupies most of its volume and is surrounded by a thin aqueous layer. (**D**) *Parasteatoda tepidariorum* glue droplet. (**E**) The same *P. tepidariorum* droplet flattened to show a smaller adhesive core within its aqueous layer. (**F**) *Latrodectus hesperus* droplet extended at 50% RH to the point of pull-off. (**G**) *Parasteatoda tepidariorum* droplet extended at 40% RH to the point of pull-off. (**H**) A droplet from the same *P. tepidariorum* individual extended at 60% RH to the point of pull off with the deflection angle *θ* of the support line indicated.

In both orb-webs and cobwebs, glue droplets delay the escape of insects giving a spider more time to subdue its prey (Eberhard 2020). Droplets in both webs are comprised of an extensible proteinaceous core that is surrounded by a hygroscopic aqueous layer that hydrates and conditions these adhesive proteins and their supporting fibers (Fig. 1A–E; Vollrath and Tillinghast 1991; Townley and Tillinghast 2013; Jain et al. 2018; Opell et al. 2018b). Currently, the identities of the proteins in the core of aggregate glue droplets are unknown. But the primary mechanism proposed to promote adhesion of aggregate glues to insect surfaces has thus far been the addition of sugar groups to the amino acid sidechains of glue proteins after translation (glycosylation) (Sahni et al. 2010, 2011; Townley and Tillinghast 2013; Amarpuri et al. 2015; Jain et al. 2015). Polysaccharides have similarly been implicated in promoting adhesion in a variety of bioadhesives (Wagner et al. 1992; Wetherbee et al. 1998; Byern and Klepal 2006; Tarakhovskaya 2014; Wang et al. 2015; Flammang et al. 2016; Huang et al. 2016). An overlooked modification to spider aggregate glue proteins is phosphorylation, even though phosphorylated serine is the most common modification found in aquatic bioadhesives (Stewart et al. 2011), and phosphate is abundant in the adhesive capture spirals of *Argiope aurantia* (Tillinghast et al. 1992).

Strong candidates for orb-web glue protein components are aggregate spidroin (AgSp) 1 and AgSp2. These two proteins are members of the spidroin (spider fibrous protein) gene family, are expressed in aggregate glands, and have the potential to be glycosylated or phosphorylated (Choresh et al. 2009; Collin et al. 2016; Stellwagen and Renberg 2019). However, these proteins have not been experimentally demonstrated to be found in glue droplets of any spider species, and the actual modifications of these proteins are unknown. Additional aggregate-expressed proteins have been described for the cobweb weaver, *Latrodectus hesperus* including aggregate silk factors (AgSFs) 1 and 2 (Vasanthavada et al. 2012) and spider coating peptides (SCPs) (Hu et al. 2007). Proteomics identified the former in the connection joints of cobwebs and the latter as coating fibers throughout the web, but none of these proteins were found in the gumfoot glue droplets.

Despite gross similarities in molecular composition, there are notable differences in the bioadhesives of orb-webs and cobwebs. The adhesive droplets of orb-webs are supported by a pair of flagelliform fibers, which are found nowhere else in the web, whereas cobweb adhesive droplets are supported by one to four fibers, which are most likely major ampullate fibers like those forming the upper irregular thread network of a cobweb and the frame and radial threads of an orb-web (Blackledge et al. 2005; Boutry and Blackledge 2008; Greco and Pugno 2021). An orb-web’s capture spiral thread is suspended between adjacent radial threads and remains anchored to these after adhering to an insect (Meyer et al. 2014; Greco et al. 2019). The connection of the capture spiral to the radial lines allows the radial lines to dissipate the kinetic energy of flying prey that hit the capture spiral (Sensenig et al. 2012). In contrast, each of a cobweb’s adhesive threads extends downward from an irregular thread network and is attached to a substrate by a pyriform silk disk (Fig. 1A; Wolff et al. 2015; Wolff and Gorb 2016). Regularly spaced glue droplets are found along the entire length of an orb-web’s capture thread, whereas they are found only on the bottom 5–15 mm of a cobweb capture thread (Fig. 1A; Eberhard et al. 2008). Orb-webs capture flying prey, whereas cobwebs predominantly capture crawling prey. When an insect contacts one or more gumfoot lines, the line’s weak pyriform anchor releases. Elastic energy stored in the gumfoot lines, and probably also in threads of the web’s upper tangle, pull the attached prey from the surface, depriving the insect of an attachment with which to more easily pull free of the gumfoot line (Blackledge et al. 2005; Argintean et al. 2006; Sahni et al. 2011; Greco and Pugno 2021).

The difference in method of prey capture is also associated with differences in the performance of orb-web and cobweb adhesive threads. After an orb-web capture spiral adheres to an insect, its flagelliform fibers and the adhesive proteinaceous glue both extend as the insect struggles to escape. This extension causes the thread to assume a robust suspension bridge configuration that sums the adhesion of multiple glue droplets and dissipates energy generated by the struggling prey (Opell and Hendricks 2007, 2009; Sahni et al. 2010, 2011; Guo et al. 2018, 2019). In contrast, although the viscosity of gumfoot proteins must be low enough to spread and establish adhesive contact, they must also be stiff enough to remain firmly attached to an insect’s surface. Because only the upper end of the gumfoot line is anchored, droplet extension would contribute little to prey retention. Likewise, after the gumfoot line’s pyriform attachment releases, the thread’s strength rather than its extensibility is important for retaining prey (Argintean et al. 2006). These differences lead us to hypothesize that the proteinaceous core of gumfoot glue droplets should be stiffer (have a greater elastic [Young’s] modulus) than that of orb-web aggregate glue droplets. As the material properties of only orb-web proteinaceous glue droplets have been characterized, we tested this hypothesis by determining the elastic modulus and toughness of the gumfoot line glue droplets of two members of the family Theridiidae, the western black widow spider *L. hesperus* Chamberlin and Ivie, 1935 and the common house spider *Parasteatoda tepidariorum* (Koch, 1841).

Orb-web capture threads are environmentally responsive because their droplets’ hygroscopic aqueous layer causes both droplet volume and adhesive protein performance to track ambient humidity. However, the glue droplets of *L. hesperus* showed very little change in elasticity and adhesion across a 75% relative humidity (RH) range (Sahni et al. 2011; Jain et al. 2016). The droplets of *L. hesperus* contain a greater percentage of proteinaceous material than do the droplets on most orb-web weaving species capture lines (Sahni et al. 2011; Opell et al. 2013). Consequently, this small aqueous volume may be incapable of significantly increasing the total water content of a glue droplet. Therefore, another objective of our study was to determine if a lack of humidity responsiveness is characteristic of another member of the family Theridiidae. We investigated this by extending the glue droplets of *P. tepidariorum* at two humidities and constructing stress–strain curves from which we determined the elastic modulus and toughness of the species’ adhesive proteins.

To complement measuring material properties of gumfoot glue droplets, we identified the protein components and their post-translational modifications (PTMs) using proteomics of the gumfoot line with and without glue. This work provides the first experimental evidence for any protein components in the aggregate glue droplets of araneoid spiders. Our integrated molecular and biomechanics approach allows us to infer the attributes of protein components that confer interfacial adhesion as well as those that confer cohesiveness, the ability to resist crack propagation between two glued surfaces. We are also able to relate the differences in humidity responsiveness of house spider aggregate droplets compared with black widow droplets to species-specific differences in protein sequence, PTMs, and the non-protein components of the glue droplets.

## Materials and methods

### Biomechanical properties of adhesives

#### Thread collection and preparing

We used procedures and instrumentation employed previously to examine the properties of orb-web adhesive (Opell et al. 2019; Opell and Stellwagen 2019) to characterize the material properties of gumfoot adhesive on threads spun by seven adult female *L. hesperus* collected near Riverside, CA and seven adult female *P. tepidariorum* collected near Lexington, VA. Spiders were housed individually in small (24.6 × 15.2 × 16.8 cm) Lee’s Kritter Keepers® with a black construction paper insert around its perimeter that could be lifted out. This ensured that newly spun gumfoot lines could be identified and easily collected.

Individual gumfoot lines were collected on the tips of forceps that were blocked open to accommodate the 4800 µm spacing of supports glued to a microscope slide sampler to which we transferred them (Opell et al. 2011). Double sided carbon tape (Cat #77816; Electron Microscope Sciences, Hatfield, PA, USA) on both the tips of the forceps and support edges ensured that a line’s native tension was maintained when, after adhering to the forceps, we cut it free from the web with iris scissors. After securing threads to the sampler, we slid droplets away from a central droplet in each 4800 µm long gumfoot line span using the tip of a wooden applicator stick sharpened to expose a few xylem fibers wetted with distilled water. This allowed the tip of a probe used to extend a droplet to contact only the central droplet.

**Fig. 2:**
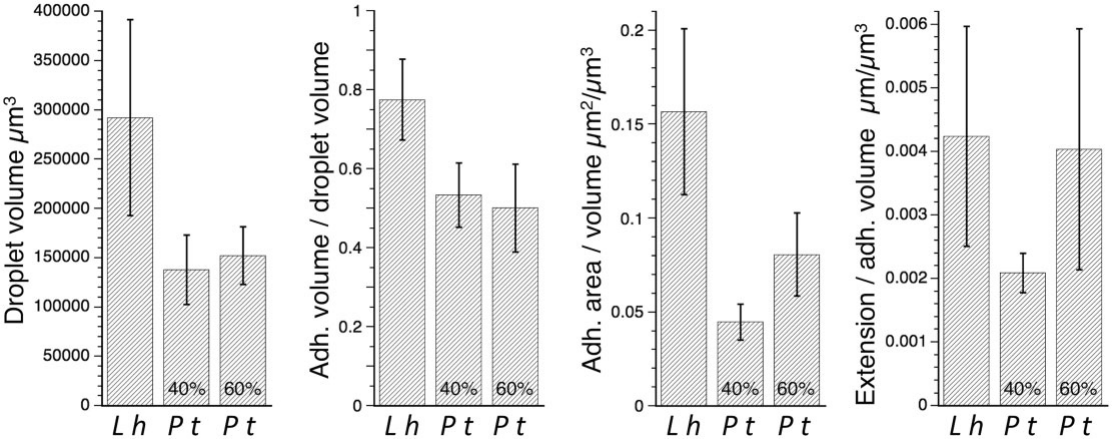
Gumfoot glue droplet adhesive (Adh.) properties that are independent of support line deflection. Extension per adhesive volume is reported for all species at 80% full extension to account for the observation that force and stress on a *L. hesperus* droplet decreases near the end of its extension (Fig. 3). Error bars are ±1 standard error.

Two lines of evidence indicate that this procedure did not remove the aqueous layer that covered the droplets glue core. When measuring flattened droplets, as described below, we were able to distinguish the glue core from a surrounding aqueous layer. As shown in ***Fig. 2***, when droplets of *P. tepidariorum* were characterized at 40% and 60% RH the surface areas and extensions of their adhesive increased in a manner consistent with the presence of an outer hygroscopic aqueous layer. Thus, although we cannot rule out the possibility that a small portion of the aqueous layer was removed, a droplet was contained within an aqueous layer during its extension.

#### Characterization of adhesive core volume

The adhesive core of theridiid’s glue droplets can be visualized more clearly after a droplet has been flattened. Therefore, we photographed a suspended droplet both prior to extension and again after it had been extended and flattened, as described below (Fig. 1B–E). We computed a suspended droplet’s volume (DV) from its length (DL, dimension parallel to the support line) and width (DW), as measured with ImageJ (Rasband http://www.uhnresearch.ca/facilities/wcif/imagej/ 2006), using the formula (Liao et al. 2015):

$$\mathrm{DV}=\frac{\left(2\pi \times {\mathrm{DW}}^{2}\times \mathrm{DL}\right)}{15}.$$

We flattened a droplet while it was still at test humidity within the observation chamber described below. We dropped a clean glass cover slip onto the suspended droplet from a magnetically triggered device attached to the underside of the chamber’s glass cover. An image of the flattened droplet allowed us to measure the surface area of both the droplet and of its adhesive core. Dividing a DV by its surface area yielded droplet thickness. Multiplying the surface area of the adhesive core by droplet thickness yielded adhesive core volume.

Amorphous proteins are present in the aqueous layer of orb web glue droplets (Amarpuri et al. 2015) and presumably also in the aqueous layer of gumfoot glue droplets. However, the degree to which these proteins contribute mechanically to a droplet’s extension is unknown. Because the methods described above do not allow us to visualize or quantify these proteins, we based our calculations of a droplet’s adhesive volume on the adhesive core that we can visualize. Although this may underestimate a droplet’s adhesive volume, this approach seems preferable to significantly overestimating adhesive volume by assuming it to be equal to droplet volume.

#### Glue droplet extension

In preparation for droplet extension, we placed a microscope slide sampler into a glass-covered, humidity-controlled chamber that rested on the mechanical stage of a Mitutoyo FS60 inspection microscope (Mitutoyo America Corp., Aurora, IL, USA). An adjustable port on the side of the chamber allowed us to insert a probe and align its 413 µm wide, polished steel tip (cleaned before each test with 100% ethanol on a Kimwipe_**^®^**_) with a droplet before anchoring the probe’s extending shaft. We then used the microscope stage’s *X* axis manipulator to bring the probe’s tip into contact with the droplet before advancing the probe an additional 250 µm to securely adhere the droplet to the probe. Finally, we activated a stepping motor attached to the stage manipulator’s *X*-axis, which moved the glue droplet’s support line away from the probe at a velocity of 69.6 µm s^-1^ while the droplet’s extension (Fig. 1F–H) was captured in a 60-frame per second video made with a Canon T1i digital camera. Examples of these videos are provided in Supplementary Materials.

During these procedures, temperature was maintained at 23^°^C by a thermostat-controlled, Peltier thermo-electric module attached to the observation chamber. A Fisher Scientific^®^ Instant Digital Hygrometer, whose tip extended through the chamber wall, monitored humidity. We established humidity by using a small dish of silica gel desiccant to lower humidity or a small dish containing a piece of distilled water-moistened Kimwipe_**^®^**_ to raise humidity. We made small adjustments to humidity by gently blowing into a tube connected to the chamber through a cylinder containing distilled water-saturated cotton to raise humidity and by drawing room air into the chamber to lower humidity. The ability of this technique to precisely control humidity is reported elsewhere (Opell et al. 2013).

#### Adhesive material property determination

We chose to construct true stress–true strain curves for droplet adhesive because engineering stress–strain curves, which are typically used for very stiff materials, assume that cross sectional area (CSA) of a material does not change as force is applied. In contrast, true stress–true strain curves, which are typically used for more plastic materials, account for the instantaneous CSA of a material as force is applied. It was clear from our videos of extending glue droplets that adhesive CSA did change during extension, violating the assumption of engineering stress–strain curves. Therefore, true stress–true strain curves seemed more appropriate because they allowed us to more fully account for the quantifiable features of glue droplets and their performance.

True stress–true strain curves also allowed us to account for inter-species, inter-individual, and inter-humidity differences in the volume of adhesive within a droplet. We did this by quantifying the adhesive volume directly from the actual droplet that was extended. This was important in gumfoot droplets because their size along an individual’s sticky line was sometimes less uniform than is typical for an orb-web weaver’s capture thread. In *P. tepidariorum* this also allowed us to account for any humidity-related change in adhesive volume between 40% and 60% RH test humidities.

During tests, the lengths of a gumfoot lines were fixed at 4800 µm, and our methods assume that the support fiber diameters did not change during extension or, in *P. tepidariorum*, between humidities. Although we could not measure the diameters of the support lines directly when they were being tested, the change in diameter due to fiber elongation would have been very small. At maximum droplet extension the mean length increase of each 2400 µm side of the foundation line supporting a droplet was very small: 17 µm (0.7%) in *L. hesperus*, 94 µm (3.9%) in *P. tepidariorum* at 40% RH and 91 µm (3.8%) in *P. tepidarorum* at 60% RH. Therefore, we do not believe that these changes compromised our determination of adhesive properties.

Two features distinguish the extension of gumfoot glue droplets from orb-web capture spiral glue droplets. Late in their extension some orb-web droplets transition to a second phase. In Phase 1 extension, the adhesive filament remains surrounded by the aqueous material, as it does during the suspension bridge configuration of a capture spiral (Opell and Hendricks 2009). Phase 2 extension is characterized by the formation of tiny drops of aqueous solution that expose portions of the lengthening adhesive filament (Opell et al. 2018a; Opell and Stellwagen 2019). All gumfoot glue droplet extensions were Phase 1 (Fig. 1F–H).

A second difference is related to the proportion of adhesive proteins in a glue droplet. At 55% RH, the mean adhesive volume of five orb-web weaving species averaged 29% of droplet volume (Opell et al. 2017, 2019), whereas in *L. hesperus* and *P. tepidariorum* adhesive volume averaged 69% of droplet volume. The size of a droplet’s core affects the perceived length of the adhesive cylinder between the thread’s support line and the probe to which a droplet adheres when droplet extension is initiated. Orb-web glue droplets begin to extend when this space is approximately the diameter of the droplet’s adhesive core. Therefore, studies of orb-web droplets have used the diameter of a droplet’s core when configured as a sphere to compute an adhesive filament’s CSA for the purpose of determining the true stress on a droplet at the initiation of extension and in computing true strain during extension (Opell et al. 2018a; Opell and Stellwagen 2019). However, given the greater proportion of adhesive in a gumfoot droplet, it appears that extension begins when the space between the support line and probe approximates that of the adhesive core’s radius when configured as a sphere. Although adhesive core radius appears the better value to use in computing theridiid adhesive properties, we also computed elastic modulus and toughness using core diameter to document the effects of using both values.

We viewed droplet extension movies with iMovie_**^®^**_ 10.1.4 (Apple Inc., Cupertino, CA, USA), dividing the elapsed time between the initiation of droplet extension and droplet pull-off into 20% intervals. At the initiation of extension and at each of the five subsequent intervals we measured droplet length and the angular deflection, *θ*, of its support line (Fig. 1F–H) using an on-screen Onde Rulers_**^®^**_ 1.13.1 (Ondesoft Computing, Inc., Beijing, China). Each extended droplet was initially situated at the center of a 4800 µm long gumfoot strand. Therefore, we used the support line’s deflection angle in conjunction with the number of fibers comprising the support line, their diameters, and their elastic moduli (Supplementary Table S1) to determine the force that each side of the support line exerted on the extending droplet. We then resolved these force vectors to determine the force perpendicular to the probe on the adhesive filament within a droplet. Formulas used for these calculations are reported in the literature (Opell et al. 2018a).

**Fig. 3:**
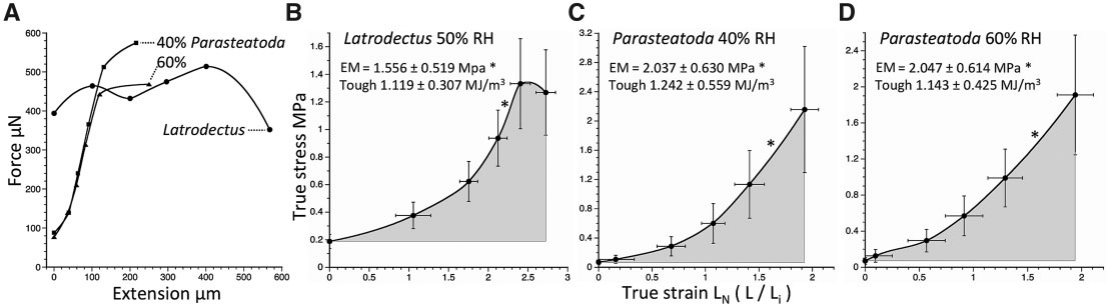
Force—extension (**A**) and true stress–true stain curves (**B–D**) of the two study species. In each figure, points represent the mean values for all measured individuals at 20% extension intervals. In B–D, error bars are ±1 standard error. Asterisks identify the portion of the curves from which elastic modulus was determined.

True stress on an extending adhesive filament was determined by dividing the force pulling a droplet from the probe by adhesive filament CSA. At the initiation of extension, we determined CSA by dividing adhesive core volume by adhesive length, determined as either the radius or the diameter of the core when configured as a sphere. At each of the five remaining extension intervals, we determined CSA by dividing core volume by measured droplet length. True strain is reported as the natural log of the droplet length divided by its initial length (radius or diameter of the adhesive core when configured as a sphere). We computed elastic modulus from the slope of the linear portion of a true stress–true strain curve: 60–80% extension intervals for *L. hesperus* and 80–100% intervals for *P. tepidariorum* (***Fig. 3***). Adhesive toughness was determined as the area under this curve, computed as a series of rectangles whose sides were defined by extension intervals and whose heights were set to the mean true stress of the bounding intervals. Because a droplet’s adhesive was under stress prior to extension, we subtracted from this total area the area of a narrow rectangle defined by the initial true stress on a droplet and total droplet true strain.

There are no data on the responses of aqueous material-coated flagelliform fibers to changes in humidity, although these fibers become stiffer when their aqueous coat is removed (Guinea et al. 2010). Furthermore, the axial fiber of cribellate silk, which is likely homologous to flagelliform silk, becomes more extensible with increasing humidity (Piorkowski et al. 2021). More relevant to the gumfoot lines of cobweb weavers, very high humidity (>75%) causes major ampullate fibers of many spiders species, including our target species, to supercontract (Boutry and Blackledge 2010). In addition, *L. hesperus* and *P. tepidariorum* gumfoot lines are slightly more extensible than the dry portion of these fibers (Blackledge et al. 2005; Boutry and Blackledge 2008). It is thus possible that when the aqueous material is diluted by the absorption of atmospheric moisture as RH increases that the elastic modulus of these fibers may decrease, allowing them to be deflected with less force. If this occurred in *P. tepidariorum* threads, the adhesive elastic modulus computed at 40% RH might be underestimated and that computed at 60% RH overestimated. The effect would be to make the differences that we report at these humidities conservative. We address this issue in two ways. We characterized glue droplets and their adhesives in ways that are not dependent on support line deflection (Fig. 2) and we modeled the effect of humidity on *P. tepidariorum* adhesive properties by increasing support line elastic modulus by 10% at 40% RH and reducing elastic modulus by 10% at 60% RH.

#### Statistical analyses

Statistical analyses were performed with JMP (SAS Institute, Cary, NC, USA). The normality of data was evaluated with Anderson–Darling tests, with *P*≥0.05 considered normally distributed. We compared normally distributed values with two-tailed *t*-tests and non-normally distributed value with Wilcoxon/Kruskal–Wallis chi-square tests, in both cases considering differences with *P*≤0.05 significant. Unless otherwise indicated, values are reported as mean ± 1 standard error.

### Protein composition and PTMs

#### Gumfoot line collection

We obtained *L. hesperus* as penultimate females in or near Denver, CO, Las Vegas, NV, and Phoenix, AZ, USA in late September 2016, and as adult females from Riverside, CA, USA in Fall 2018. We collected penultimate and adult female *P. tepidariorum* from Lexington, VA, USA in June 2017 and 2018. We housed all spiders in plastic deli containers (7 cm deep, 10 cm diameter across the bottom, 12 cm diameter across the top) with black cardboard inserts. We fed spiders crickets three times per week during weeks in which gumfoot lines were not collected. We collected gumfoot lines for protein analysis only from adult females (penultimates were allowed to mature before starting collection). Once gumfoot line collection began, spiders always received at least one week of feeding in between 5-day long collection periods.

We moved spiders to Kritter Keepers as described above for gumfoot line collection for protein analysis. Spiders were never fed in these containers in order to minimize insect contamination of the glue droplets. We collected gumfoot lines on E-shaped cardboard cutouts (1.9 cm between lines of E) with a piece of double-sided tape on the middle and top lines of the E, but not the bottom. The gumfoot glue naturally adhered to the bottom line. We cut the top of the gumfoot line’s axial fiber to avoid collecting the tangle portion of the web.

We combined gumfoot lines of multiple individuals on a single collector over 3–5 consecutive days. For *L. hesperus* caught in 2016, gumfoot lines of 24 individuals were combined during three separate weeks. For *P. tepidariorum* caught in 2017, gumfoot lines of 20 individuals were combined during three separate weeks. The three collection times represent the three samples in “Run 1” below. For spiders collected in 2018, *L. hesperus* was divided into two groups of individuals (8 and 10 individuals), and *P. tepidariorum* was divided into three groups (9, 10, and 11 individuals). Gumfoot lines were combined for individuals within a group. In order to obtain sufficient material for protein processing, we had to combine collections from multiple separate weeks (but same group of individuals).

At the end of the 3–5 consecutive day collection period, the gumfoot lines were cut away from the E-shaped cardboard collector such that the axial fiber with glue droplets (hereafter “Glue+Fiber”) was separated from the axial fiber without glue droplets (hereafter “Fiber”). The two portions were separately stored in sterile 1.5 mL centrifuge tubes (Fisher Brand, ThermoFisher Scientific, Waltham, MA, USA) at −80_**°**_C until solubilization.

#### Protein purification, tryptic digests, and mass spectrometry

We incubated 1–3 mg of silk in glass vials containing 0.5–2 mL hexafluoroisopropanol (AlfaAesar, Tewksbury, MA, USA) overnight at room temperature, with vortexing at the beginning and end of incubation. Almost all silk solubilized under these conditions, although the higher mass samples sometimes retained visible white pieces. Two 10% aliquots were moved to 1.5 mL tubes. The original vial and the aliquots were allowed to dry at room temperature in a fume hood. Dried silk was stored at −20_**°**_C until further processing.

One of the 10% aliquots was used to estimate protein abundance, and one was used for visualization with Sodium Dodecyl Sulfate–Polyacrylamide Gel Electrophoresis (SDS–PAGE). To estimate protein abundance, the dried silk was resuspended in 6 µL 8 M guanidine hydrochloride (GdnHCl) and then diluted to 0.8 M GdnHCl with TE (10 mM Tris–HCl, pH 7.5, 1 mM EDTA). Bovine serum albumin (BSA) was similarly resuspended in 0.8 M GdnHCl, TE and diluted to 1000, 750, 500, 250, 125, 50, and 25 ng/µL. Duplicate 25 µL aliquots of silk samples and BSA standards were mixed with 200 µL of BCA working reagent (Pierce™ BCA Protein Assay Kit, ThermoFisher Scientific) and incubated for 30 minutes at 37°C or at least 2 hours at room temperature. The absorbance at 562 nm was measured with an Infinite M1000Pro (TECAN, Seestrasse, Mannedorf, Switzerland).

For visualization with SDS-PAGE, the second 10% aliquot was resuspended in 25 mM Tris, 10% glycerol, 5 mM MgCl_2_, 2% SDS, 150 mM NaCl, denatured by incubating at 95_**°**_C for 20 min with 10% beta-mercaptoethanol, and placed on ice. Assuming the same amount of protein in this aliquot as in the one used for the BCA assay, we loaded 5–10 µg of protein on 8–12% polyacrylamide gels. Proteins were electrophoresed at 120 V for ∼1 h, washed, and stained with Bio-Safe Coomassie (Bio-Rad, Hercules, CA, USA) or SimplyBlue™ Safe Stain (ThermoFisher Scientific), and imaged under white light (ChemiDoc™ MP, Bio-Rad).

To prepare silk samples for tryptic digests and mass spectrometry, we resuspended dried silks in 60 µL 8 M GdnHCl, 3.2 µL 100 mM dithiothreitol (Invitrogen Brand, ThermoFisher Scientific), and denatured at 95°C for 20 min. Denatured proteins were moved to ice, then to room temperature, and then alkylated with 15 mM iodoacetamide (Acros Organics, Fair Lawn, NJ, USA). We diluted the GdnHCl concentration to 1 M with the addition of 50 mM ammonium bicarbonate and 1 mM CaCl_2_. Assuming eight times as much protein in the stock sample as the 10% aliquot used for BCA, we digested 5–10 µg protein with 2.5–5 µg trypsin (Promega, Madison, WI, USA) overnight at 37°C. Peptides were then desalted by adding 10% trifluoroacetic acid (Fisher Brand, ThermoFisher Scientific) to digested peptides to achieve a pH < 3 and passing through a C18 ZipTip_**^®^**_ (Millipore, Burlington, MA, USA), washing with 0.1% TFA, and eluting with 0.1% TFA, 50% acetonitrile. Cleaned peptides were dried with a speed vacuum and shipped on ice to the University of Arizona Proteomics Core (Phoenix).

LC–MS/MS analysis was done on a Q Exactive Plus mass spectrometer (Thermo Fisher Scientific) at the University of Arizona Proteomics Core. Peptides were eluted from an Acclaim PepMap 100 trap column onto an Acclaim PepMap RSLC analytical column (both from Thermo Fisher Scientific). Chromatography was performed using a 5–20% gradient of solvent B (acetonitrile, 0.1% formic acid) over 90 min, then 20–50% solvent B for 10 min, 50–95% solvent B for 10 min, 95% solvent B for 10 min, and 5% solvent B for 10 min. Solvent A was 0.1% formic acid in water. Flow rates were 300 nL/min using a Dionex Ultimate 3000 RSLCnano system (ThermoFisher Scientific), and peptides were ionized with an EasySpray nanoESI source. Data dependent scanning was performed by the Xcalibur software (Andon et al. 2002) using a survey scan at 70,000 resolution scanning mass/charge (353–1550) followed by higher-energy collisional dissociation tandem mass spectrometry at 27 normalized collision energy of the 10 most intense ions. Dynamic exclusion was set to place any selected mass/charge peaks on an exclusion list for 30 s after a single MS/MS.

#### Protein identification, label-free quantitation, and PTMs

Initial searches for proteins and PTMs (Ser/Thr/Asn glycosylation and Ser/Thr/Tyr phoshorylation) were performed by the University of Arizona Proteomics Core with Thermo Proteome Discoverer (ThermoFisher Scientific). We supplied databases of protein sequences based on a transcriptome for *L. hesperus* (Clarke et al. 2015) and the genome for *P. tepidariorum* (Schwager et al. 2017) with gene predictions that incorporated silk gland expression information (Haney et al. 2019). We added sequences based on cDNA clones for Major Ampullate Spidroin (MaSp) 1 (MH367500.1), MaSp2 (MH367501.1), Minor Ampullate Spidroin (MiSp) (KX584055.1, KX584022.1), and AgSF2 (JZ979955.1, JZ979956.1) to the *P. tepidariorum* protein set because the encoding sequences were not well represented in the genome. For further searches with MaxQuant (Cox and Mann 2008), we generated a reduced database of proteins based on a combination of all proteins identified by the Proteomics Core (combined run 1, run 2, no modifications, and modifications) for each species. These reduced protein databases included 188 protein sequences for *L. hesperus* and 279 for *P. tepidariorum*.

We completed label-free quantitation (LFQ) with MaxQuant on the two MS runs separately. Default settings were used for the LFQ runs. We searched for PTMs (both Ser/Thr/Asn glycosylation and Ser/Thr/Tyr phosphorylation) using raw files from both runs simultaneously for each species. We searched for variable modifications to allow detection of both unmodified and modified peptides.

#### Statistical comparisons to identify “glue” proteins

The LFQ results of runs 1 and 2 were consolidated based on identified proteins or protein clusters—groups of proteins that could not be distinguished based on peptides from the MS. For further analysis, we retained proteins that had a MaxQuant score >50 in at least one run in the LFQ search or were identified as modified in five of the Glue+Fiber samples in the PTM search (e.g., all Glue+Fiber samples for *L. hesperus*; at least five of six for *P. tepidariorum*) and had a score >50 in the PTM search. We further required proteins to have an LFQ > 0 in at least two samples from each run. We thus feel confident we have identified high-quality proteins consistently present in gumfoot lines.

**Fig. 4:**
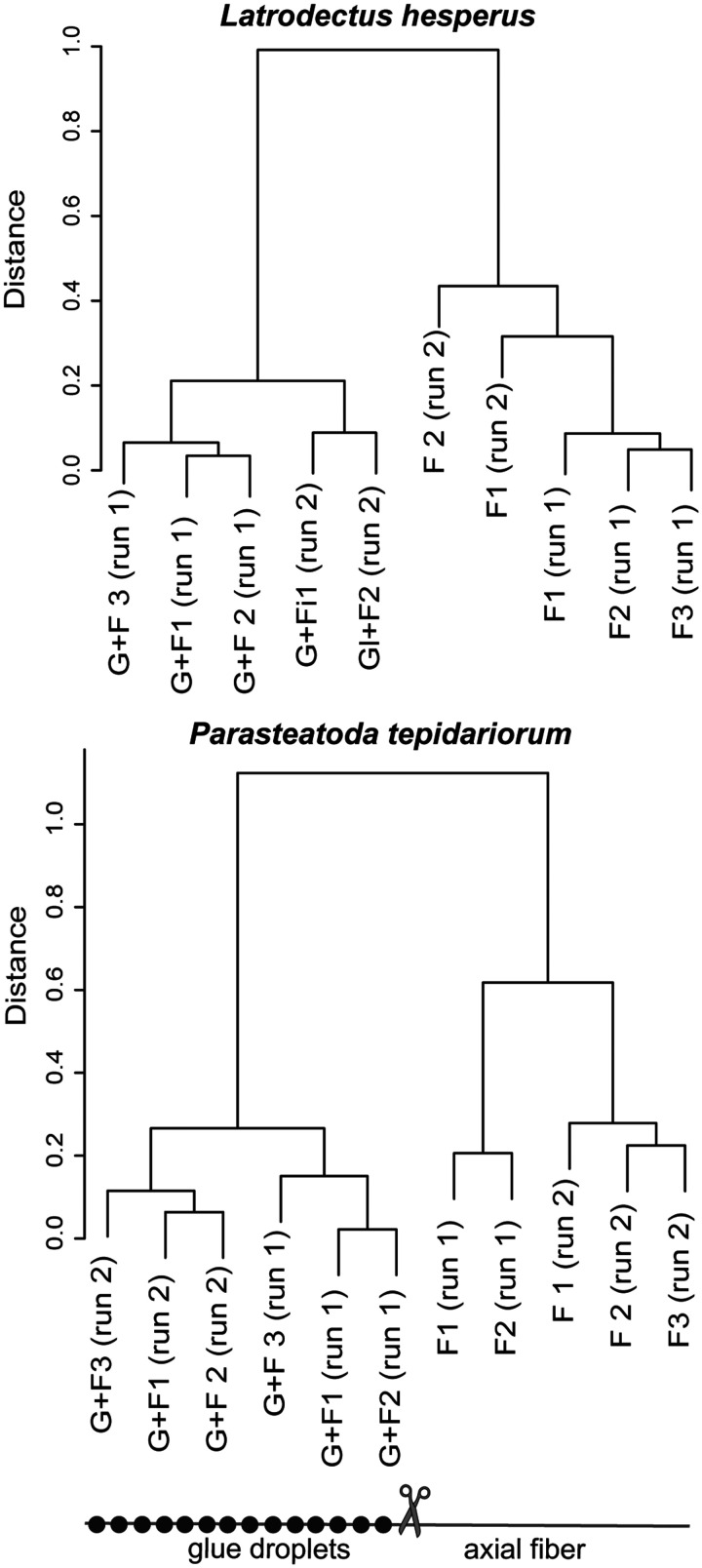
Hierarchical clustering of silk samples based on LFQ of proteins identified with mass spectrometry. Gumfoot fibers covered with glue droplets (Glue+Fiber, “G + F”) were separated from the top portion of the gumfoot fiber that is not covered in glue (Fiber, “F”). Two mass spectrometry runs (run 1 versus run 2) were completed for each species (see the “Materials and methods” section). Hierarchical clustering was based on pairwise distance (one-Spearman’s correlation coefficients) of the LFQ for the high confidence proteins in each sample.

We calculated pairwise Spearman correlation coefficients among all samples based on the LFQ of each protein. Because we found that Glue+Fiber samples were well correlated and distinct from Fiber samples even across the two mass spec runs (Fig. 4), we combined runs for further statistical analysis. One of the Fiber samples in *P. tepidariorum* had low quality scores for most proteins and did not correlate well with any other samples. This sample was removed prior to further analysis (but see Supplementary Table S2 for LFQ values for this sample and *t*-test results with and without inclusion of this sample).

For each protein, we calculated the relative difference (Tusher et al. 2001) in LFQ between Fiber and Glue+Fiber samples. We identified proteins significantly more abundant (based on LFQ) in Glue+Fiber than Fiber samples using a one-tailed Student’s *t*-test implemented in R (R Core Team 2020). We then estimated the false discovery rate (FDR) with the Benjamini and Hochberg method (Benjamini and Hochberg 1995).

#### Expression and annotation of gumfoot proteins

The expression values of the *L. hesperus* transcripts were obtained from Clarke et al. (2017) which compared the Clarke et al. (2015) transcriptome to 23 mRNA libraries from 10 tissues (including seven silk gland types, venom glands, cephalothoraxes, and all silk glands combined) using RSEM (Li and Dewey 2011) with the default parameters. Expression levels for *P. tepidariorum* were obtained from comparisons of the Haney et al. (2019) gene set to 24 mRNA libraries from seven silk gland types using RSEM. RNA sequencing for *P. tepidariorum* involved the same methods for RNA isolation as Clarke et al. (2017) but RNA samples were sent to Novogene (Sacramento, CA, USA) for library preparation and sequencing.

We calculated the differential expression between the aggregate and non-aggregate glands and between the anterior and posterior aggregate glands using DESeq2 version 1.28.1 (Love et al. 2014). We annotated the two gene sets by BLAST (Altschul et al. 1990) comparisons to multiple databases including manually curated databases of spider-specific genes shown in Supplementary Table S3 (AgSps with tBlastX, Spidroin Terminal Regions with BlastP, and Additional UniProt Silk Genes with BlastX), and more taxonomically inclusive databases (SwissProt [The UniProt Consortium 2021] with BlastP and PFAM [Mistry et al. 2021] with Hmmer version 3.2.1).

## Results

### Material properties of gumfoot droplets

Supplementary Table S1 reports the material properties of the two species’ gumfoot foundation lines, Supplementary Table S4 reports features of their gumfoot droplets, and Supplementary Table S5 reports droplet extension values. The stress–strain curves (Fig. 3), and the elastic modulus and toughness values derived from them, use the radius of a droplet’s adhesive when configured as a sphere to determine both the stress on a droplet at the initiation of extension and the true strain on a droplet. Substituting the adhesive core’s diameter for radius did not affect the elastic modulus of either species but reduced *L. hesperus* toughness by 9% and *P. tepidariorum* toughness at each humidity by 4%.

Both the volume of *L. hesperus* glue droplets and their proportion of adhesive is greater than that of *P. tepidariorum* glue droplets (Fig. 2). The greater flattened adhesive surface area per adhesive volume of *L. hesperus* (Fig. 2) is consistent with the lower elastic modulus of this species’ adhesive, as is this species’ greater extension per adhesive volume when compared with that of *P. tepidariorum* adhesive measured at 40% RH (Fig. 3B–D). Force–extension curves reveal that force on extending *P. tepidariorum* droplets increases rapidly and markedly during extension, whereas the force on *L. hesperus* droplets is already great when extension begins and changes less during extension (Fig. 3A). Only late in extension does force on *P. tepidariorum* droplets at 40% RH exceed that at 60% RH.

**Fig. 5:**
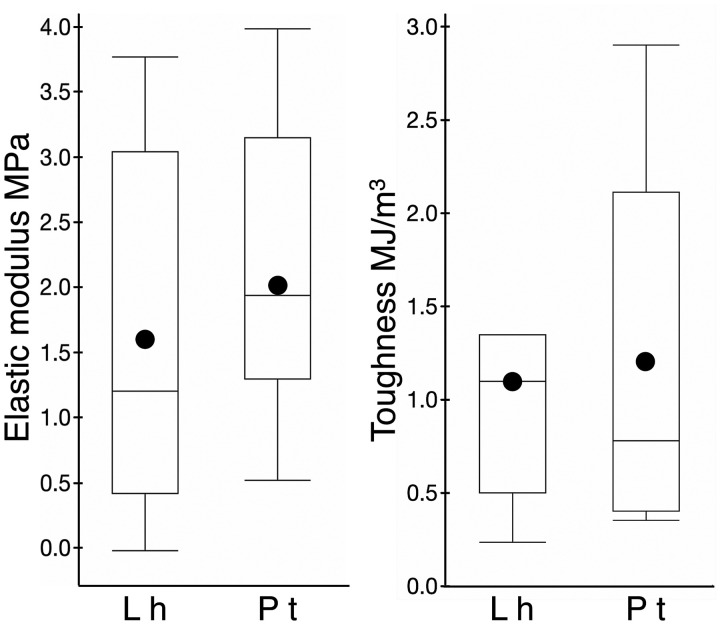
Box plots comparing the elastic modulus and toughness of *L. hesperus* (Lh) and *P. tepidariorum* (Pt). Values of *L. hesperus* were determined at 50% RH, and of *P. tepidariorum* are means of individual values determined at 40% and 60% RH. The dot within each box denotes the mean value, the line denotes the median value, the top of the box denotes the 75% quartile, and the bottom of the box denotes the 25% quartile.

True stress–true strain curves show that at both 40% and 60% RH *P. tepidariorum* adhesive is under greater stress throughout extension than is *L. hesperus* adhesive, although *L. hesperus* adhesive sustained greater strain (Fig. 3B–D). The elastic modulus of *P. tepidariorum* adhesive decreased as humidity increased from 40% to 60% and, at both humidities, was greater than that of *L. hesperus* adhesive. The elastic modulus and toughness of *L. hesperus* adhesive were normally distributed (*P*** **=** **0.373 and 0.319, respectively). The elastic modulus of *P. tepidariorum* adhesive was normally distributed at 40% RH, 60% RH, and when each individual’s values were averaged for the two RH (*P*** **=** **0.306, 0.260, and 0.647, respectively). The toughness of *P. tepidariorum* adhesive was normally distributed at 60% RH and when toughness at 40% and 60% were averaged (*P*** **=** **0.126 and 0.658, respectively), but not at 40% RH (*P*** **=** **0.003). The mean elastic modulus of *P. tepidariorum* adhesive (2.042_** ± **_0.445 MPa) exceeded that of *L. hesperus* adhesive (1.556 ± 0.519 MPa) (Fig. 5), but this difference was not significant (one-tailed *t*-test, *P*** **=** **0.246). Likewise, the toughness of *L. hesperus* adhesive (1.119 ± 0.307 MJ/m^3^) did not differ significantly from the averaged *P. tepidariorum* values (1.192 ± 0.361 MJ/m^3^) (one-tailed *t*-test *P*** **=** **0.440) (Fig. 5). Despite increases in both the area and extension of *P. tepidariorum* adhesive relative to adhesive volume as humidity increased (Fig. 2), neither the elastic modulus nor the toughness of this species’ adhesive differed significantly between 40% and 60% RH (one-tailed *t*-test, *P*** **=** **0.496 and Wilcoxon *P*** **=** **0.8480, respectively).

Increasing the elastic modulus of *P. tepidariorum* gumfoot support lines by 10% at 40% RH and reducing this value by 10% at 60% RH did not affect the computed elastic modulus because this only changed the position of the stress–strain curve relative to its *Y* axis, and therefore, did not change the curve’s slope from which adhesive elastic modulus was determined. However, adhesive toughness changed slightly with 40% values being 1.346 ± 0.615 MJ/m^3^ and 60% values being 1.028 ± 0.382 MJ/m^3^. However, this greater difference in toughness (cf., Fig. 3C and D) was not significant (Wilcoxon *P*** **=** **0.655).

### Gumfoot glue protein composition and quantitation

We identified 105 proteins (or protein clusters) that were consistently found in the gumfoot lines of *L. hesperus* and 80 proteins (or protein clusters) that were consistently found in the gumfoot lines of *P. tepidariorum* (Supplementary Tables S2 and S6). Protein clusters are similar proteins encoded by distinct genes (as designated by the Trinity assembly for *L. hesperus*, or the genome assembly for *P. tepidariorum*) that could not be distinguished based on peptides in the mass spectrometry. We refer to protein clusters as proteins for the remainder of the manuscript. Using LFQ for these proteins, we found that the samples derived from the portion of the gumfoot line with glue (Glue+Fiber) correlated well with each other, even across two batches of animals and MS runs (Fig. 4). They were also distinct from the portion of the gumfoot line comprised of only major ampullate axial fibers (Fiber), which were well-correlated with each other (Fig. 4).

**Fig. 6:**
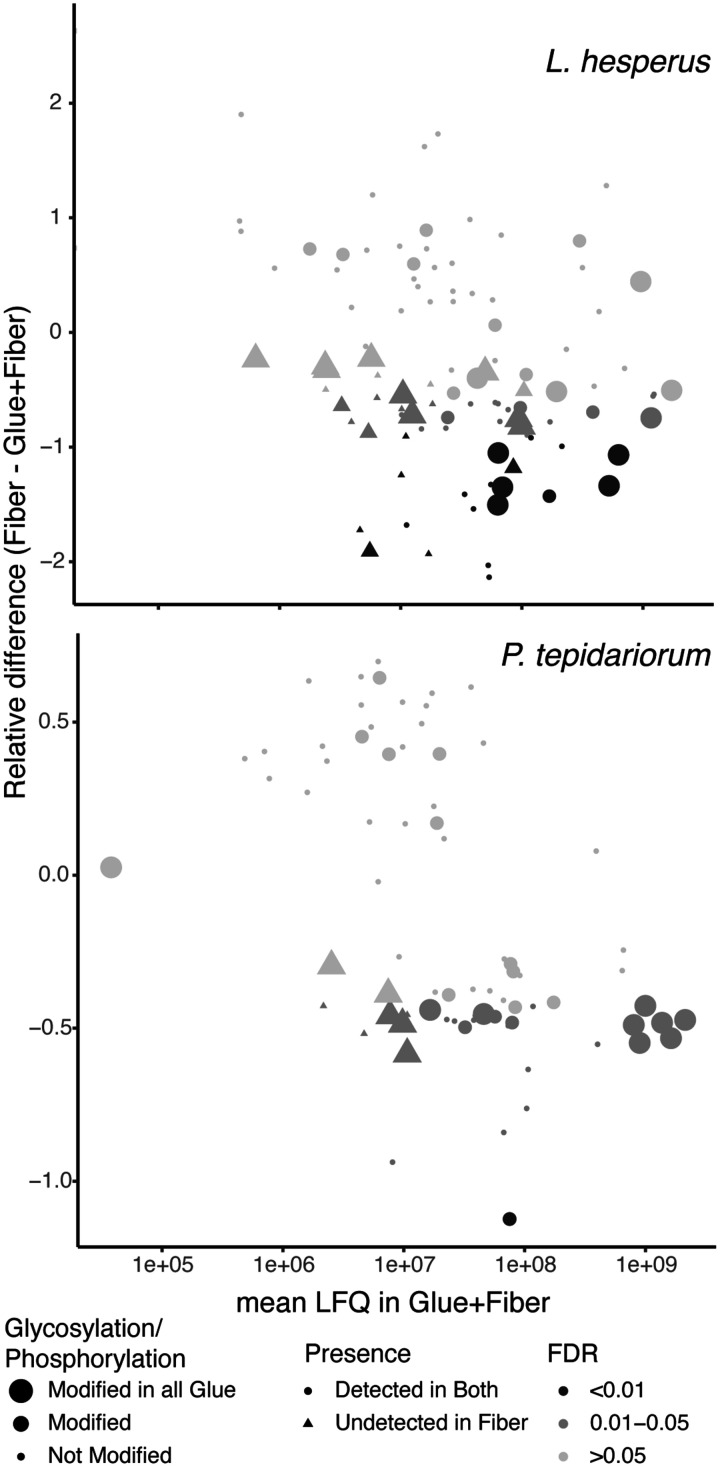
Mean LFQ of proteins in gumfoot Glue+Fiber samples versus relative difference between Fiber LFQ and Glue+Fiber LFQ. Negative relative difference (Tusher et al. 2001) values indicate enrichment in Glue+Fiber. One-tailed *t*-tests were used to identify proteins more abundant in Glue+Fiber than in Fiber samples; the FDR (Benjamini and Hochberg 1995) for each protein is indicated by the darkness of the dot/triangle. Protein modifications are indicated by the size of the dot. Triangles indicate proteins that were not detected in Fiber.

In *L. hesperus* we detected 77 proteins in both Glue+Fiber and Fiber samples, 26 in just Glue+Fiber, and 2 in just Fiber. In *P. tepidariorum* we detected 70 proteins in both Glue+Fiber and Fiber samples, and 10 in just Glue+Fiber (***Fig. 6***). Gel images confirm multiple proteins in Glue+Fiber samples not found in Fiber (Supplementary Fig. S1). Because the axial fiber runs throughout the entire gumfoot line, we expected fibrous proteins to be detected in both sample types. Proteins that were detected in only Glue+Fiber samples are likely to aggregate glue proteins. However, limiting glue protein candidates to those only found in Glue+Fiber is likely too restrictive for two reasons: (1) Fiber samples may be contaminated with glue during collection, and (2) black widows are known to place small amounts of aggregate secretions on the entire fiber (e.g., SCPs; Hu et al. 2007). We thus additionally considered proteins that were significantly more abundant in Glue+Fiber than Fiber to be strong candidates for aggregate glue proteins. We found 48 such proteins in *L. hesperus* and 33 in *P. tepidariorum* (Fig. 6).

Within Glue+Fiber samples mean LFQ values of proteins ranged across five orders of magnitude (Fig. 6). The top 10 most abundant proteins included ones expected to be in major ampullate fibers: MaSp1, Cysteine Rich Protein 1 (CRP1), and CRP3 in *L. hesperus* (***Table 1***) and MaSp1 and MaSp2 in *P. tepidariorum*. MaSp2 was not detected in the gumfoot lines of *L. hesperus* (Supplementary Table S6). Also represented among the most abundant proteins in gumfoot Glue+Fiber were AgSps in both species, including fragments of AgSp1 in *L. hesperus*, and AgSp1, AgSp2, and AgSp-like proteins in *P. tepidariorum* (Table 1). AgSp2 was found in *L. hesperus* gumfoot lines but was not one of the most abundant proteins. These AgSp homologs were all significantly more abundant in Glue+Fiber than Fiber samples. In *L. hesperus*, AgSF2 had the highest LFQ in Glue+Fiber samples, although it was marginally not significantly more abundant in Glue+Fiber than Fiber (*t*-test FDR = 0.054 for enrichment in Glue+Fiber). AgSF2 had previously been undetected in gumfoot lines and was thought to form connection joints (Vasanthavada et al. 2012). Additional proteins that have never been reported from aggregate glands or silk fibers were also highly abundant in Glue+Fiber samples. Of special note are proteins predicted to include a Thyroglobulin type-1 repeat (Table 1).

**Table 1:** The most abundant proteins in gumfoot lines

LFQ rank in Glue+Fiber	*L. hesperus* annotation	*P. tepidariorum* annotation
1	AgSF2	**AgSp1**
2	**Flag C-terminal**	**AgSp-like**
3	**AgSp1 N-terminal**	**AgSp2 N-terminal**
4	**AgSp1 repetitive**	**Unknown**
5	**Thyroglobulin type-1 repeat**	**Unknown**
6	MaSp1	**AgSp2 C-terminal**
7	**AgSp1 repetitive**	MaSp1
8	**AgSp1 C-terminal**	**Thyroglobulin type-1 repeat**
9	CRP1	**Thyroglobulin type-1 repeat**
10	CRP3	MaSp2

See Fig. 6 for their mean LFQ in Glue+Fiber. Bolded proteins were significantly more abundant in Glue+Fiber than Fiber samples (FDR < 0.05).

### Gumfoot glue protein expression and annotation

**Fig. 7:**
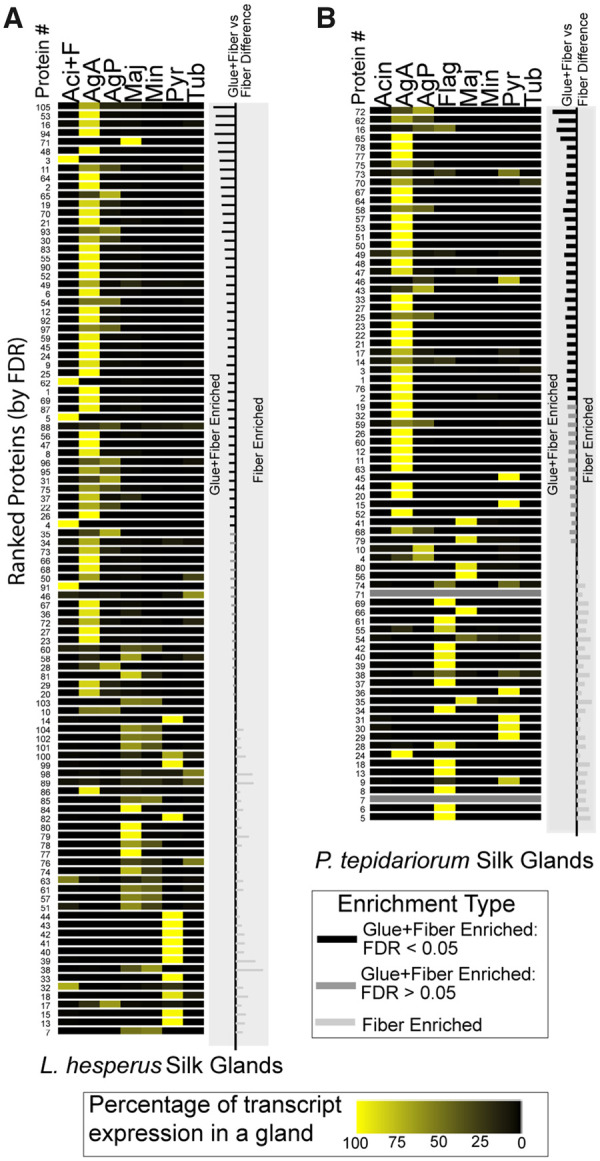
Gumfoot protein expression patterns. Expression patterns for (**A**) *L. hesperus* and (**B**) *P. tepidariorum* proteins identified with high confidence from mass spectrometry of gumfoot lines with glue (Glue+Fiber) or without glue (Fiber). The proteins are listed from top to bottom by order of their one-sided Student’s *t*-test *P*-value comparing Glue+Fiber samples to Fiber alone, with the smallest *P*-values at top. The percentage expression in each silk gland type (heatmaps) is shown for protein-encoding transcripts, using the summed RSEM Transcripts per Million Mapped transcripts across gland types reported from Clarke et al. (2017) for *L. hesperus* or from this study for *P. tepidariorum*. The column to the left of the heat maps is protein numbers that can be connected to Fig. 7 and Supplementary Tables S2 and S6. Horizontal bars to the right of heat maps show relative difference of the LFQ for each protein between Fiber and Glue+Fiber samples. Proteins more abundant in the Glue+Fiber samples are on the left half and those more abundant in Fiber samples are on the right half. The colors of the lines reflect the t-test FDRs for Glue+Fiber enrichment.

**Fig. 8:**
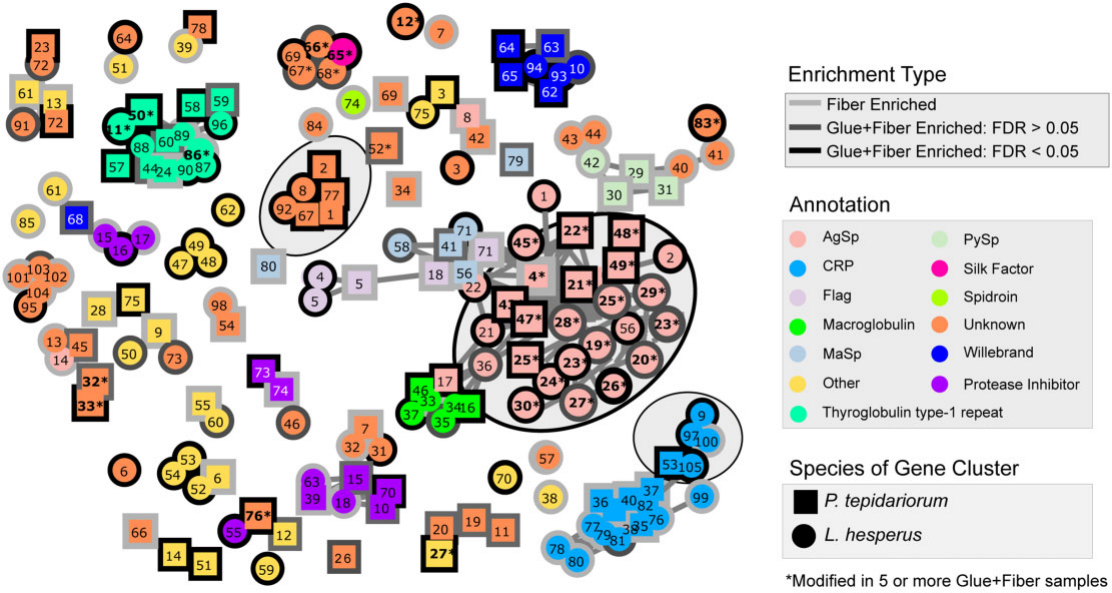
The relationships among gumfoot proteins using a network graph. The proteins are shown grouped by blastP comparisons between all the members of protein clusters (proteins that could not be distinguished by peptides in the MS) with *L. hesperus* protein clusters as circles and *P. tepidariorum* as squares with the protein cluster numbers inside (as in Fig. 7 and Supplementary Tables S2 and S6). Proteins that are modified in at least five of the Glue+Fiber samples have an asterisk. The interior of the proteins is colored by the protein annotation with the outside border showing the *t*-test classes. We have further encircled three protein groups of interest, including the Aggregate-Specific CRPs, the AgSp group, and the unknown conserved cysteine-containing protein group described in Supplementary Fig. S2.

Gumfoot glue proteins should be expressed in the aggregate glands. Indeed, proteins that were significantly more abundant, or even just more abundant in Glue+Fiber than Fiber, were almost exclusively encoded by transcripts expressed in the aggregate glands (***Fig. 7***) for both species. Intriguingly, the majority of expression for proteins enriched in Glue+Fiber was in the anterior aggregate glands, consistent with the hypothesis that the anterior aggregate glands of theridiid spiders synthesize the gumfoot glue while the posterior aggregate glands have specialized for an alternative function, spraying glue on prey items (Townley and Tillinghast 2013). In *L. hesperus*, four of the proteins enriched in Glue+Fiber are encoded by transcripts with majority expression in Aciniform+Flagelliform glands. While we cannot definitively determine which of the two gland types expressed these transcripts, two lines of evidence suggest they originate from the flagelliform glands. First, two of these proteins are fragments of flagelliform spidroins (#4 and 5 in Figs. 7A and 8), one of which was the second most abundant protein in gumfoot Glue+Fiber (Table 1). Second, in *P. tepidariorum*, none of the proteins identified in gumfoot lines had appreciable expression in aciniform glands. In contrast, *P. tepidariorum* transcripts with majority expression in flagelliform glands encoded proteins that were more abundant in Fiber samples (Fig. 7B). In both species, transcripts with majority expression in major ampullate or pyriform glands were more abundant in Fiber samples.

Major ampullate gland silk is thought to form the bulk of cobwebs, including the axial fiber of gumfoot lines (Blackledge et al. 2005). In orb-web weavers, flagelliform glands synthesize the axial fiber over which aggregate glue is spread. Our expression results suggest that *P. tepidariorum* retains this function for flagelliform glands but that *L. hesperus* may have co-opted the flagelliform glands for glue production. The flagelliform glands of *L. hesperus* are greatly reduced in size relative to *P. tepidariorum* and orb-web weavers (Clarke et al. 2017; Chaw and Hayashi 2018).

A few different gene families appear to encode glue protein components common to *L. hesperus* and *P. tepidariorum* (***Fig. 8***). In addition to the AgSps and Thyroglobulin type-1 repeat containing proteins discussed above, we found a group of CRPs (Pham et al. 2014) enriched in Glue+Fiber that were also encoded by transcripts with majority expression in aggregate glands (Figs. 7 and 8). Another family with conserved cysteine residues but without homology to any known proteins was also identified (Fig. 8, gray highlighting and Supplementary Fig. S2). Three different protein families enriched in Glue+Fiber were predicted to have protease inhibition functions, including an alpha-2-macroglobulin domain and trypsin inhibitors (Fig. 8). In contrast, Thyroglobulin type-1 repeat domains are usually thought to function in controlled proteolysis (pfam.xfam.org/family/PF00086). Another protein family found enriched in Glue+Fiber contains a von Willebrand factor type C domain (Fig. 8), which often functions in protein–protein interactions (pfam.xfam.org/family/PF15430).

### Hydrophilicity and isolelectric points of gumfoot proteins

**Fig. 9:**
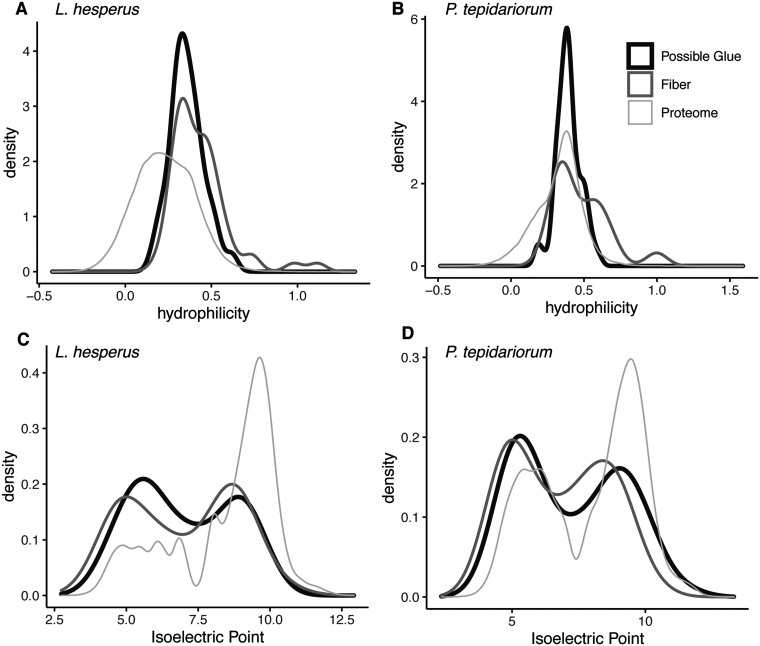
Hydrophilicity (**A, B**) and isoelectric point (**C, D**) density plots for gumfoot proteins relative to the entire proteome. Hydrophilicity was calculated by averaging hydrophobic indices for each protein (Kyte and Doolittle 1982). Isoelectric points were calculated by averaging side-chain isoelectric points as well as N and C-termini (Bjellqvist et al. 1994). Proteins identified with high confidence from the MS of gumfoot lines were treated independently even if some could not be separated by the MS (e.g., each protein in a protein cluster is counted as a separate protein in this analysis). Proteins considered to be “Possible Glue” in this figure were significantly more abundant in Glue+Fiber than Fiber samples, were modified in all the Glue+Fiber samples, or exhibited enriched gene expression in aggregate glands relative to other gland types according to DESeq2. Proteins that did not fit one of these criteria were classified as “Fiber.” The “Proteome” is the remaining set of reference proteins for each species (see the “Materials and methods” section).

Gumfoot glue droplets attract atmospheric moisture, due to the presence of organic low molecular mass components but the proteins may also vary in their attraction to water. We found that gumfoot proteins tend to be more hydrophilic than the remainder of the proteome for both *L. hesperus* (*W* = 5,429,138, *P* < 2.2e−16, Wilcoxon rank sum test) and *P. tepidariorum* (*W* = 4,186,102, *P* = 1.9e−10) (***Fig. 9***A and B). Fiber proteins were slightly more hydrophilic than “Possible Glue” proteins (as per Fig. 9) for *L. hesperus* (*W* = 2368, *P* = 0.012) and *P. tepidariorum* (*W* = 1712, *P* = 0.035); but “Possible Glue” proteins were still more hydrophilic than the proteome for *L. hesperus* (*W* = 3,556,182, *P* = 1.2e−12) and *P. tepidariorum* (*W* = 2,818,008, *P* = 5.5e−05).

Another metric of gumfoot protein composition that may be important for material properties of glue droplets is the distribution of isoelectric points. At physiological pH_**∼**_7, proteins with an isoelectric point <7 are negatively charged while those >7 are positively charged. Oppositely charged proteins will have electrostatic interactions. In both species, the distribution of isoelectric points is bimodal consistent with the presence of many oppositely charged proteins (Fig. 9C and D). Furthermore, the distribution of isoelectric points is significantly different between gumfoot proteins and the remainder of the proteome for *L. hesperus* (*W* = 17,278,533, *P*-value < 2.2e−16) and *P. tepidariorum* (*W* = 7,768,534, *P*-value = 5.1e−08). Fiber proteins and “Possible Glue” proteins (as per Fig. 9) had overlapping isoelectric points in *L. hesperus* (*W* = 1826, *P* = 0.82) and *P. tepidariorum* (*W* = 2499, *P* = 0.16).

### PTMs of gumfoot proteins

Glue+Fiber samples were highly enriched for glycosylated and phosphorylated peptides relative to Fiber samples (***Table 2***). No proteins were found to be consistently enriched in all Fiber samples, while many were consistently enriched in Glue+Fiber samples (Fig. 8).

**Table 2:** Gumfoot glue proteins are highly glycosylated and phosphorylated

Sample type	Unmodified peptides	Glycosylated peptides (% of total)	Phosphorylated peptides (% of total)	Both glycosylated and phosphorylated (% of total)
*L. hesperus* Glue+Fiber	6590	885 (11.2)	319 (4.1)	63 (0.8)
*L. hesperus* Fiber	4704	70 (1.5)	36 (0.8)	8 (0.2)
*P. tepidariorum* Glue+Fiber	11141	1087 (8.1)	1099 (8.1)	136 (1.0)
*P. tepidariorum* Fiber	3753	94 (2.4)	112 (2.8)	14 (0.4)

Fisher’s exact tests comparing modified to unmodified peptides in Glue+Fiber relative to Fiber were all significant: *L. hesperus* glycosylated versus unmodified OR = 9.01, *P *<* *2.2e−16; *L. hesperus* phosphorylated versus unmodified OR = 6.32, *P *<* *2.2e−16; *P. tepidariorum* glycosylated versus unmodified OR* *=* *3.90, *P *<* *2.2e−16; *P. tepidariorum* phosphorylated versus unmodified OR = 3.30, *P *<* *2.2e−16.

**Fig. 10:**
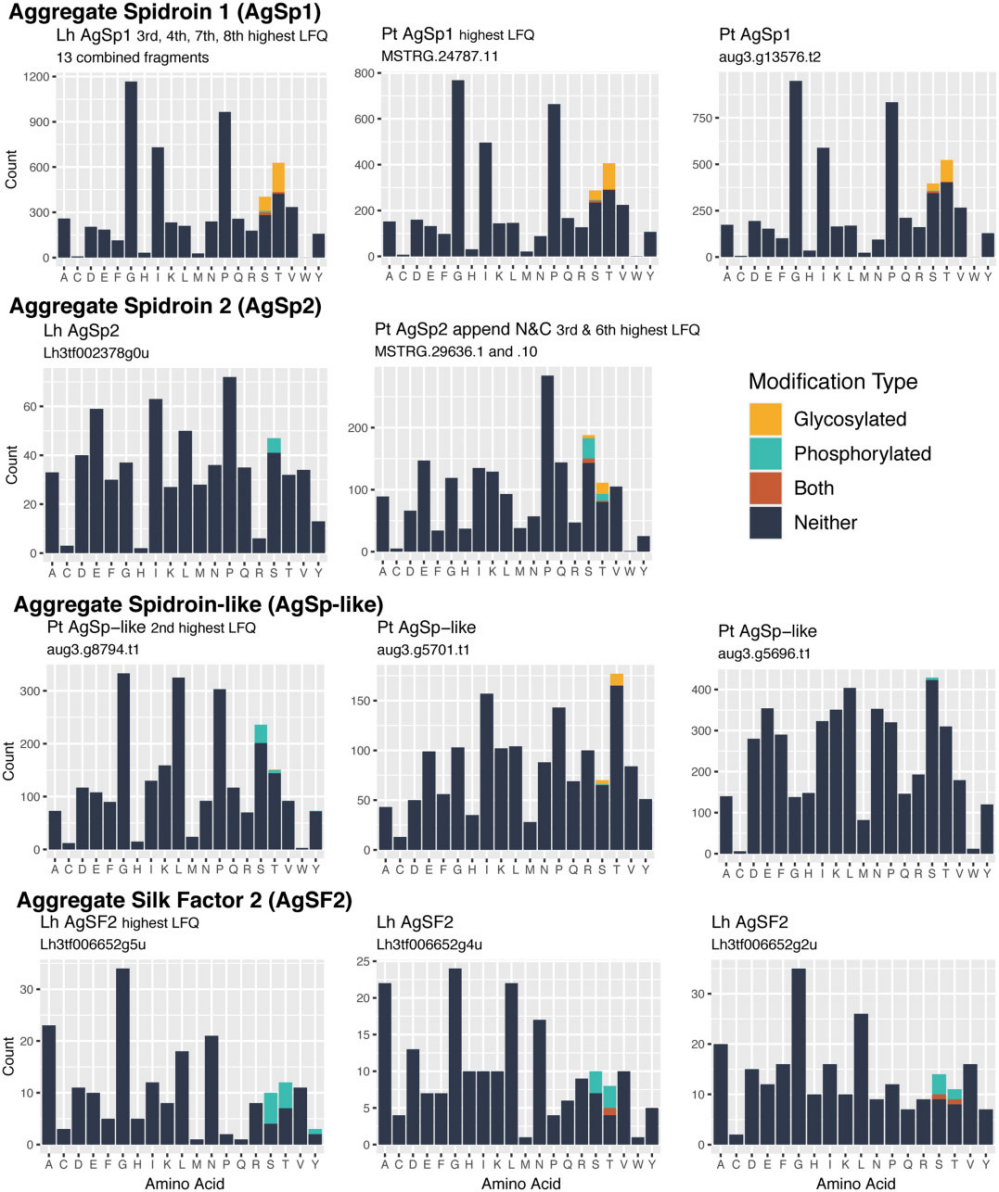
Amino acid composition plots for AgSp homologs and AgSF2 homologs that were modified in at least five Glue+Fiber samples and had more than one modified position. Glycosylated and phosphorylated amino acids are indicated by separate colors. The genome of *P. tepidariorum* has two complete AgSp1 paralogs and three complete or nearly complete AgSp-like paralogs. In contrast, AgSp2 of *P. tepidariorum* is split between an N-terminal encoding contig (two isoforms) and a C-terminal encoding contig (two isoforms). For simplicity, one N-terminal isoform was combined with one C-terminal isoform for this figure. The transcriptome of *L. hesperus* included 13 AgSp1-encoding fragments, one of which encodes the N-terminal domain and one of which encodes the C-terminal domain. The remaining fragments appear to encode different pieces of the repetitive region. Since these transcripts are likely incompletely assembled, we combined all fragments for this figure. Sequences of all AgSp homologs identified in gumfoot samples with mapped PTMs are in Supplementary Fig. S3. In addition to the three AgSF2 homologs shown here, three more homologs were identified in gumfoot samples. However, two of them were not modified in all the Glue+Fiber samples and one had only a single modified position. Complete sequences of all AgSF2 homologs identified in gumfoot samples with mapped PTMs are in Supplementary Fig. S4.

While multiple proteins with various functions were occasionally modified, only a few protein families were consistently modified in all Glue+Fiber samples and in both species (Fig. 8). We found abundant glycosylation of AgSp1 in both our species (***Fig. 10***). We additionally found many phosphorylated positions in AgSp2 of both species and an AgSp-like protein in *P. tepidariorum* (Fig. 10). Glycosylation or phosphorylation was found in conserved Ser and Thr positions of the repetitive region of AgSp1 (Supplementary Fig. S3). AgSp2 and AgSp-like homologs did not show conserved positions of modification (Supplementary Fig. S3). In *L. hesperus*, multiple homologs of AgSF2 were phosphorylated (Fig. 10). Some positions of phosphorylation were conserved among homologs, while others were sporadic (Supplementary Fig. S4).

As seen in Fig. 8, Thyroglobulin type-1 domain is the only family other than AgSp to have PTMs in both *L. hesperus* and *P. tepidariorum*. One of these *P. tepidariorum* sequences was phosphorylated at three positions; two *L. hesperus* sequences were phosphorylated at one position each. The positions of phosphorylation were not conserved among the three sequences (Supplementary Fig. S5).

## Discussion

Prior to our work, candidate aggregate glue droplet proteins had been proposed, but to our knowledge, none had been experimentally validated (Choresh et al. 2009; Vasanthavada et al. 2012; Collin et al. 2016; Stellwagen and Renberg 2019). We have provided experimental validation for proposed candidates, as well as numerous additional protein components of spider’s aggregate glue. Rather than finding a single dominant protein as in most spider silk fibers, the aggregate glue of a cobweb weaver’s gumfoot droplet is a relatively complex mixture of proteins with various functions. We also measured material properties of this proteinaceous glue. Here we describe potential relationships between protein components and two important attributes of any bioadhesive: adhesion and cohesion.

Consistent with a presumed role of polysaccharides in promoting interfacial adhesion (Sahni et al. 2010, 2011; Townley and Tillinghast 2013; Amarpuri et al. 2015; Jain et al. 2015), we found extensive glycosylation of proteins in the gumfoot glues of our two cobweb weavers (Table 2). Glycosylation was primarily found on AgSp1 in both *L. hesperus* and *P. tepidariorum*. AgSp1 also ranked among the most abundant proteins in gumfoot glues according to LFQ (Fig. 6 and Table 1). Prior to our work, the role of phosphorylation in aggregate glue adhesion had been virtually ignored, but we found extensive phosphorylation of gumfoot glue proteins (Table 2). Phosphorylated amino acids can be used to coordinate ionizable molecules on surfaces, greatly promoting adhesion (Stewart et al. 2011). Phosphate groups may also contribute to cohesion, which we discuss below. AgSp1 was occasionally phosphorylated in both species but another AgSp, AgSp2, was more extensively phosphorylated (Fig. 10). AgSp2 ranked among the most abundant proteins in *P. tepidariorum* but not in *L. hesperus* gumfoot glues (Table 1). Instead, homologs of AgSF2 were abundant and phosphorylated in *L. hesperus* (Fig. 10). These differences in composition between the two species may explain some of the differences in material properties.

**Fig. 11:**
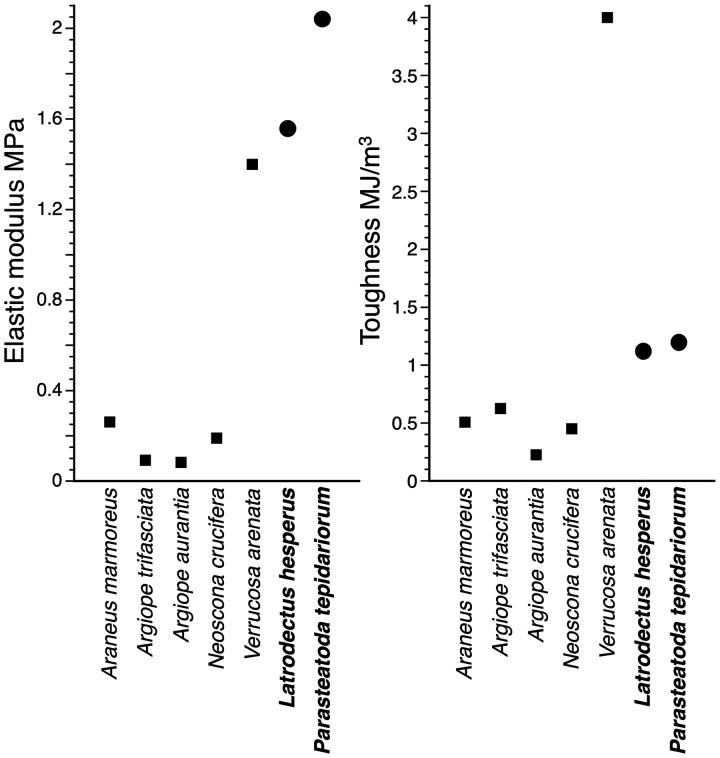
Comparison of the material properties of adhesive glue droplets of two cobweb weaving species (circles) with those of five orb-web weaving species (squares). *Latrodectus hesperus* droplets were measured at 50% RH, *P. tepidariorum* droplets were measured at 40% and 60% RH and then averaged for this figure, and values shown for orb-web weavers were measured at 55% RH (Opell et al. 2018).

The proteinaceous cores of spider aggregate glue droplets exhibit a wide range of material properties among species (***Fig. 11***). As hypothesized, the elastic modulus and toughness of gumfoot glue proteins were greater than those of most orb-web capture thread glue proteins. This is consistent with differences in each thread’s adhesive delivery system. Both the proteinaceous core and supporting flagelliform fibers of an orb-web’s capture thread must be extensible to implement a suspension bridge configuration that sums the adhesion of multiple droplets and dissipates the energy of prey struggle (Opell and Hendricks 2007, 2009; Sahni et al. 2010, 2011; Guo et al. 2018, 2019). However, because a gumfoot line detaches from a surface immediately after prey contact, stiffer glue proteins establish a firm adhesive bond and stiffer support lines resist thread extension (Blackledge et al. 2005; Wolff et al. 2015). These fibers may be able to store more energy in their initial taunt state (Argintean et al. 2006; Greco and Pugno 2021). The elastic moduli of major ampullate fibers within *L. hesperus* and *P. tepidariorum* gumfoot lines are, on average, 105 times greater than those of the five orb-web weaving species’ flagelliform fibers (Supplementary Table S7). When the number and diameters of these support lines are considered, 8.5 and 2.4 times more energy is required to extend a meter length of *L. hesperus* and *P. tepidariorum* gumfoot lines, respectively, than is required to extend the same length of an orb-web’s capture thread (Supplementary Table S7).

The proteinaceous core’s elastic modulus of the orb weaver *Verrucosa arenata* is unusual, in that it falls within the range of values for *L. hesperus* and *P. tepidariorum* gumfoot glue proteins, and the toughness of *V. arenata* glue exceeds that of gumfoot glue (Fig. 11). Flagelliform fibers of *V. arenata* are also 12.25 times stiffer than those of other orb-web weaving species that have been characterized and require an average of 1.6 times more energy to extend (Supplementary Table S7). These differences suggest that *V. arenata* capture threads are functioning differently than those of other orb-web weavers. The orb-webs of *V. arenata* are radii poor relative to their capture area, the area between a web’s inner and outer capture spiral turns (Supplementary Fig. S6). This results in their webs having much longer capture spiral spans between radii than do the webs of other species, potentially limiting cooperation among multiple threads. Furthermore, the long distance between radii may limit the ability of connection joints to transfer energy from capture spirals to radial lines (Meyer et al. 2014; Greco et al. 2019). In contrast to other orb-web weaving species for which radial lines absorb an order of magnitude more energy than the capture spirals when impacted by a flying object, *V. arrenata* radial lines absorb a similar amount of energy as the spiral lines (Sensenig et al. 2012). To compensate, *V. arenata* capture threads may have shifted toward those of gumfoot lines, which are not held taunt after prey contact.

While we do not have protein components for orb-web glue droplets, multiple attributes of the protein components we describe for gumfoot glue droplets could contribute to their cohesiveness, which we have measured as stiffness. For instance, multiple protein families were found in both species that contain conserved cysteine residues, including a number of aggregate-specific CRPs (Pham et al. 2014) and a newly discovered conserved protein family, both of which were highly enriched in gumfoot Glue+Fiber relative to Fiber (Fig. 8 and Supplementary Fig. S2). Cysteines form disulfide bonds within and among polypeptides. These covalent bonds should contribute to strong interactions within the glue droplet promoting cohesion. We also found a protein family predicted to contain a type C von Willebrand domain, which has conserved functions in forming multi-protein aggregates. Von Willebrand domains are found in blood-clotting factors and may promote the proteolysis of proteins to become enzymatically or structurally functional. In barnacle cement, a transglutaminase generates peptides that then promote protein aggregation to form the cement (Dickinson et al. 2009; So et al. 2016). The von Willebrand domain-containing proteins we found may have similar action, although it is unclear that the type C domain promotes protein–protein interactions through proteolysis. We also found Thyroglobulin Type-1 repeats in both species’ gumfoot glues. This domain often functions in controlled proteolysis (Lenarcic et al. 1997), and could regulate proteolysis by another enzyme such as the von Willebrand domain to have a similar role to barnacle glue transglutaminase—essentially to generate peptides that promote protein–protein aggregation.

Another attribute of the gumfoot glue proteins that likely contributes to cohesion, and thus stiffness, is the presence of multiple oppositely charged proteins in both species (Fig. 9C and D), which likely participate in electrostatic interactions. The addition of negatively charged phosphates to the negatively charged AgSp2, and in the case of *L. hesperus*, AgSF2, may promote the interaction of these proteins with the positively charged repetitive regions of AgSp1. Even when focusing solely on the gumfoot fiber, the proteins have a similar bimodal distribution further suggesting a role of electrostatic interactions in fiber integrity as well as in fiber-glue cohesion. The glue that pulls away from the fiber would be of little use in prey capture. An orb-web weaver’s glue droplet can adhere, extend, and pull off through many cycles without its adhesive core becoming detached from the thread’s supporting flagelliform fibers (Kelly et al. 2019).

The elasticity and adhesion of *L. hesperus* adhesive changed little over a 15–90% RH range (Sahni et al. 2011). Although we did not demonstrate a significant difference in the elastic modulus of *P. tepidariorum* adhesive protein cores during a 20% RH increase, the relative surface area and extension of this species adhesive increased with humidity (Fig. 2). The different response to humidity between *L. hesperus* and *P. tepidariorum* may be explained by differences in: (1) The composition of the two species’ glue proteins, (2) composition of the aqueous layer, (3) amount of aqueous material, or (4) a combination of these factors. We first consider protein composition. In many ways the gumfoot glue protein components of *L. hesperus* and *P. tepidarioru*m are conserved. Multiple protein families were found enriched in gumfoot Glue+Fiber relative to Fiber in both species (Fig. 8) and the most abundant proteins for both species included AgSp1 and Thyroglobulin type-1 repeats. However, the two species differed in the presence of AgSp2 and an AgSp-like protein as abundant phosphorylated proteins in *P. tepidariorum* versus AgSF2 homologs as the abundant phosphorylated proteins in *L. hesperus*. AgSF2 has many runs of Glycine that could contribute to the extensibility of *L. hesperus* glue droplets relative to *P. tepidariorum* (Vasanthavada et al. 2012). However, it is not immediately apparent how these differences would lead to *P. tepidariorum* glue droplets humidity responsiveness and not *L. hesperus*. Furthermore, both species’ glue proteins have similar hydrophilicity and a bimodal distribution of isoelectric points (Fig. 9). Electrostatic interactions in the glue droplets should become weaker as the proteins are diluted by water. This mechanism may in part explain the increased extensibility (lower cohesiveness) of orb-web and *P. tepidariorum* glue droplets with higher RH. It is possible that *L. hesperus* experiences enzymatic cross-linking of glue proteins that prevents a change in extensibility after polymerization, although we did not find evidence of enzymes unique to *L. hesperus*.

Given conserved protein sequences between the species, PTMs may play a role in their different responses to humidity. *Latrodectus hesperus* had a higher percentage of peptides that were glycosylated compared with *P. tepidariorum* (12% versus 9%). Sugar polymers can form elaborate structures, and in some cases sugar polymers can exclude water reducing their responsiveness to humidity (Hatakeyama et al. 2016). More importantly, protein-ligand interactions can also be regulated by glycosylation, and a von Willebrand factor family member has its shear stress response regulated by glycosylation (Nowak et al. 2012). In all cases, the composition of the sugar polymers as well as the linkage between individual sugars plays a role in how sugar polymers respond to both humidity and mechanical stress.

We can also compare the proportion of the aqueous material in each of the species’ glue droplets (Supplementary Table S8). As previously noted, gumfoot droplets have a smaller proportion of aqueous material than do the glue droplets of most orb-web capture threads (Opell et al. 2019). The proteinaceous core within a *L. hesperus* and *P. tepidariorum* droplet occupies, on average, 69% of the DV, as compared to an average of 29% of the volume of orb-web glue DV. However, the droplets of *P. tepidariorum* have 2.7 times more aqueous volume than do those of *L. hesperus*. If the composition of the two species’ aqueous layers is similar, this difference alone may explain the greater humidity responsiveness of *P. tepidariorum* droplets, as their larger aqueous volumes would confer a greater capacity to attract atmospheric moisture. However, glue droplets of the orb weaver *Argiope trifasciata*, which have nearly as much proteinaceous core volume as do those of *L. hesperus* (Supplementary Table S8), are very responsive to humidity (Opell et al. 2019). Therefore, compositional differences in the aqueous layers of the two theridiid species’ glue droplets cannot be ruled out.

Given the considerable difference in stiffness of glue droplets between our two cobweb weavers’ and those of four orb-web weavers, we predict protein composition of orb-web glue droplets may differ dramatically from gumfoot glue droplets. AgSps remain likely candidates as important components of orb-web glue droplets, as we have seen in our cobweb weavers. We already know that the repeat unit of AgSp1 in orb-web weavers is longer than our two cobweb weavers, with more potential positions for glycosylation and a slightly higher proportion of Gly and Pro, amino acids associated with extensibility in spider silk fibers (Stellwagen and Renberg 2019). We suspect that orb-web glue droplets will lack some of the protein attributes that we believe contribute to stiffness in gumfoot glue droplets, such as multiple cysteine-rich proteins, a strong bimodal distribution of isoelectric points, and multiple proteins that contribute to protein–protein aggregation.

In conclusion, we have provided the first experimental evidence for any proteins in the aggregate glue droplets of araneoid spiders. Cobweb weaver’s gumfoot glue droplets are stiffer and tougher than orb-web glue droplets, which can be explained by multiple attributes of protein composition. Aggregate glue proteins are highly enriched for glycosylation and phosphorylation, a previously overlooked modification. Both types of modifications likely confer adhesion, and the latter may additionally contribute to cohesion of glue proteins. While black widow glue droplets do not respond to humidity, the house spiders’ droplets become more extensible with increased humidity, similar to orb-web glue droplets. Differences in enzymatic cross-linking of proteins, PTMs, aqueous volume, or aqueous content between the black widow and other species may explain variation in humidity responsiveness. Future work will test our predictions regarding molecular correlates of glue function by expanding to a broad taxonomic sampling of araneoid spiders.

## Data availability

The data underlying this article are available in the article, its Supplementary Material, and public databases. Specifically, the mass spectrometry proteomics data have been deposited to the ProteomeXchange Consortium via the PRIDE (Perez-Riverol et al. 2019) partner repository with the dataset identifier PXD025685, and the RNA-seq data have been deposited to NCBI’s short read archive under BioProject PRJNA704711.

## Supplementary Material

icab086_Supplementary_Data

## Notes

From the symposium “The biology of sticky: Adhesive silk, fiber, and glue biomaterials across Eukaryota” presented at the virtual annual meeting of the Society for Integrative and Comparative Biology, January 3–7, 2021.
