# Supplementary material for: Protein Composition and Associated Material Properties of Cobweb Spiders’ Gumfoot Glue Droplets
Source: Integr Comp Biol. 2021 May 18;61(4):1459–80. doi: 10.1093/icb/icab086 (PMC8631074; doi:10.1093/icb/icab086)
Supplement: icab086_Supplementary_Data [file icab086_Supplementary_Data.zip › icb-2021-0105-File015.docx]

Table S1. Major ampullate thread properties (mean ± 1 sstandard error). *Young’s modulus from (Blackledge et al. 2005).

| Species | *Latrodectus hesperus* | *Parasteatoda tepidariorum* |
| --- | --- | --- |
| Number of threads | 2 (N = 5) or 4 (N = 2) | 4 (N = 7) |
| Thread diameter μm | 5.27 ± 0.36 (N = 7) | 2.29 ± 0.22 (N = 7) |
| Young’s Modulus GPa | * 3.7 ± 0.5 (N = 25) | 1.4399 ± 0.2036 (N = 8) |

Table S4. Glue droplet features (mean ± 1 standard error). Sample size each species 7.

| Species | *Latrodectus hesperus* | *Parasteatoda tepidariorum* | |
| --- | --- | --- | --- |
| % Relative humidity | 50% | 40% | 60% |
| Droplet length μm | 60 ± 20 | 28 ± 6 | 31 ± 6 |
| Droplet width μm | 44 ± 15 | 21 ± 4 | 23 ± 4 |
| Droplet volume μm^3^ | 292,158 ± 99,340 | 136,615 ± 29,671 | 152,207 ± 29,277 |
| Droplet area μm^2^ | 43,281 ± 18,296 | 4,663 ± 485 | 9,980 ± 2811 |
| Glycoprotein area μm^2^ | 31,027 ± 15,914 | 2,334 ± 366 | 4,608 ± 1,270 |
| Glycoprotein thickness μm | 9.1 ± 1.9 | 28.3 ± 5.7 | 23.1 ± 7.8 |
| Glycoprotein volume μm^3^ | 243,382 ± 95,538 | 68,610 ± 11,276 | 87,244 ± 26,536 |

Table S5. Droplet extension characteristics (mean ± 1 standard error). Sample size each species 7.

| Feature | % Droplet Extension | | | | | |
| --- | --- | --- | --- | --- | --- | --- |
|  | 0% | 20% | 40% | 60% | 80% | 100% |
| Extension μm |  |  |  |  |  |  |
| *Latrodectus hesperus* | 35 ± 5 | 101 ± 11 | 200 ± 21 | 296 ± 41 | 401 ± 68 | 568 ± 106 |
| *Parasteatoda tepidariorum* 40% RH | 24 ± 1 | 39 ± 6 | 63 ± 9 | 91 ± 12 | 131 ± 21 | 216 ± 30 |
| *Parasteatoda tepidariorum* 60% RH | 24 ± 3 | 36 ± 5 | 59 ± 8 | 83 ± 12 | 120 ± 14 | 250 ± 62s |
| Glyco Filament CS Area μm^2^ |  |  |  |  |  |  |
| *Latrodectus hesperus* | 5,687 ± 1,549 | 3,610 ± 2,223 | 1,209 ± 515 | 784 ± 290 | 587 ± 214 | 391 ± 120 |
| *Parasteatoda tepidariorum* 40% RH | 2,649 ± 303 | 2,011 ± 426 | 1,142 ± 170 | 756± 93 | 541 ± 73 | 325 ± 46 |
| *Parasteatoda tepidariorum* 60% RH | 2,938 ± 672 | 2,817 ± 954 | 1,811 ± 622 | 1,297± 447 | 846 ± 280 | 406 ± 137 |
| Axial deflection angle degrees |  |  |  |  |  |  |
| *Latrodectus hesperus* | 167 ± 2 | 166 ± 1 | 166 ± 1 | 165 ± 1 | 165 ± 1 | 167 ± 1 |
| *Parasteatoda tepidariorum* 40% RH | 168 ± 3 | 162 ± 3 | 158 ± 3 | 154 ± 3 | 151 ± 3 | 149 ± 3 |
| *Parasteatoda tepidariorum* 60% RH | 165 ± 2 | 162 ± 3 | 158 ± 4 | 155 ± 4 | 152 ± 5 | 152 ± 5 |
| True stress MPa |  |  |  |  |  |  |
| *Latrodectus hesperus* | 0.094 ± 0.025 | 0.376 ± 0.096 | 0.623 ± 0.145 | 0.937 ± 0.203 | 1.332 ± 0.326 | 1.269 ± 0.310 |
| *Parasteatoda tepidariorum* 40% RH | 0.035 ± 0.018 | 0.109 ± 0.055 | 0.287 ± 0.131 | 0.600 ± 0.271 | 1.134 ± 0.463 | 2.158 ± 0.862 |
| *Parasteatoda tepidariorum* 60% RH | 0.035 ± 0.019 | 0.126 ± 0.071 | 0.295 ± 0.124 | 0.569 ± 0.220 | 0.989 ± 0.321 | 1.911 ± 0.664 |
| Strain Δ L / Li |  |  |  |  |  |  |
| *Latrodectus hesperus* | 0 | 1.091 ± 0.250 | 1.791 ± 0.133 | 2.159 ± 0.133 | 2.441 ± 0.144 | 2.761 ± 0.134 |
| *Parasteatoda tepidariorum* 40% RH | 0 | 0.352 ± 0.181 | 0.872 ± 0.129 | 1.266± 0.095 | 1.604 ± 0.123 | 2.120 ± 0.103 |
| *Parasteatoda tepidariorum* 60% RH | 0 | 0.366 ± 0.225 | 0.837 ± 0.242 | 1.184 ± 0.243 | 1.563 ± 0.226 | 2.211 ± 0.210 |

Table S7. Comparison of the energy required to extend the support lines of orb web capture spiral and cob web gumfoot lines. The toughness of *P. tepidariorum* is the mean of 40% and 60% RH values. Values of orb weavers were computed at 55% and is from (Opell et al. 2018, 2019).

| Species | Total gumfoot foundation line cross sectional area µm^2^ | Elastic modulus  GPa | Gumfoot  foundation line toughness MJ/m^3^ | Energy of extension 1 m thread Joules |
| --- | --- | --- | --- | --- |
| *Latrodectus*  *hesperus* | 53.2 | 3.7 | 1.5639 | 83.20 |
| *Parasteatoda*  *tepidariorum* | 17.4 | 1.44 | 1.3381 | 23.28 |
| *Araneus*  *marmoreus* | 22.7 | 0.005 | 0.5077 | 11.52 |
| *Argiope*  *aurantia* | 36.2 | 0.009 | 0.2245 | 8.13 |
| *Argiope*  *trifasciata* | 13.2 | 0.008 | 0.6252 | 8.25 |
| *Neoscona*  *crucifera* | 14.1 | 0.01 | 0.4766 | 6.72 |
| *Verrucosa*  *arenata* | 3.5 | 0.098 | 4.0461 | 14.16 |

Table S8. Comparison of the adhesive volumes of orb web and cob web glue droplets. Values of *L. hesperus* were determined at 50% RH and those of *P. tepidariorium* are the means of 40% and 60% values. Values of orb weaving species were measured at 55% RH (Opell et al. 2018, 2019).

| Species | Total droplet volume µm^3^ | Adhesive volume µm^3^ | % Adhesive volume |
| --- | --- | --- | --- |
| *Latrodectus*  *hesperus* | 292,158 | 243,382 | 83 |
| *Parasteatoda*  *tepidariorum* | 144,411 | 77,927 | 54 |
| *Araneus*  *marmoreus* | 81,329 | 30,942 | 38 |
| *Argiope*  *aurantia* | 71,139 | 8,625 | 12 |
| *Argiope*  *trifasciata* | 44,414 | 34,078 | 77 |
| *Neoscona*  *crucifera* | 13,378 | 1,642 | 12 |
| *Verrucosa*  *arenata* | 10,561 | 821 | 8 |

**References**

Blackledge TA, Swindeman JE, Hayashi CY. 2005. Quasistatic and continuous dynamic characterization of the mechanical properties of silk from the cobweb of the black widow spider Latrodectus hesperus. J Exp Biol 208:1937–49.

Opell BD, Burba CM, Deva PD, Kin MHY, Rivas MX, Elmore HM, Hendricks ML. 2019. Linking properties of an orb-weaving spider’s capture thread glycoprotein adhesive and flagelliform fiber components to prey retention time. Ecol Evol 9:9841–54.

Opell BD, Clouse ME, Andrews SF. 2018. Elastic modulus and toughness of orb spider glycoprotein glue. PLOS ONE 13:e0196972.
