# Supplementary material for: Protein Composition and Associated Material Properties of Cobweb Spiders’ Gumfoot Glue Droplets
Source: Integr Comp Biol. 2021 May 18;61(4):1459–80. doi: 10.1093/icb/icab086 (PMC8631074; doi:10.1093/icb/icab086)
Supplement: icab086_Supplementary_Data [file icab086_Supplementary_Data.zip › icb-2021-0105-File020.docx]

**Figure S1**

**
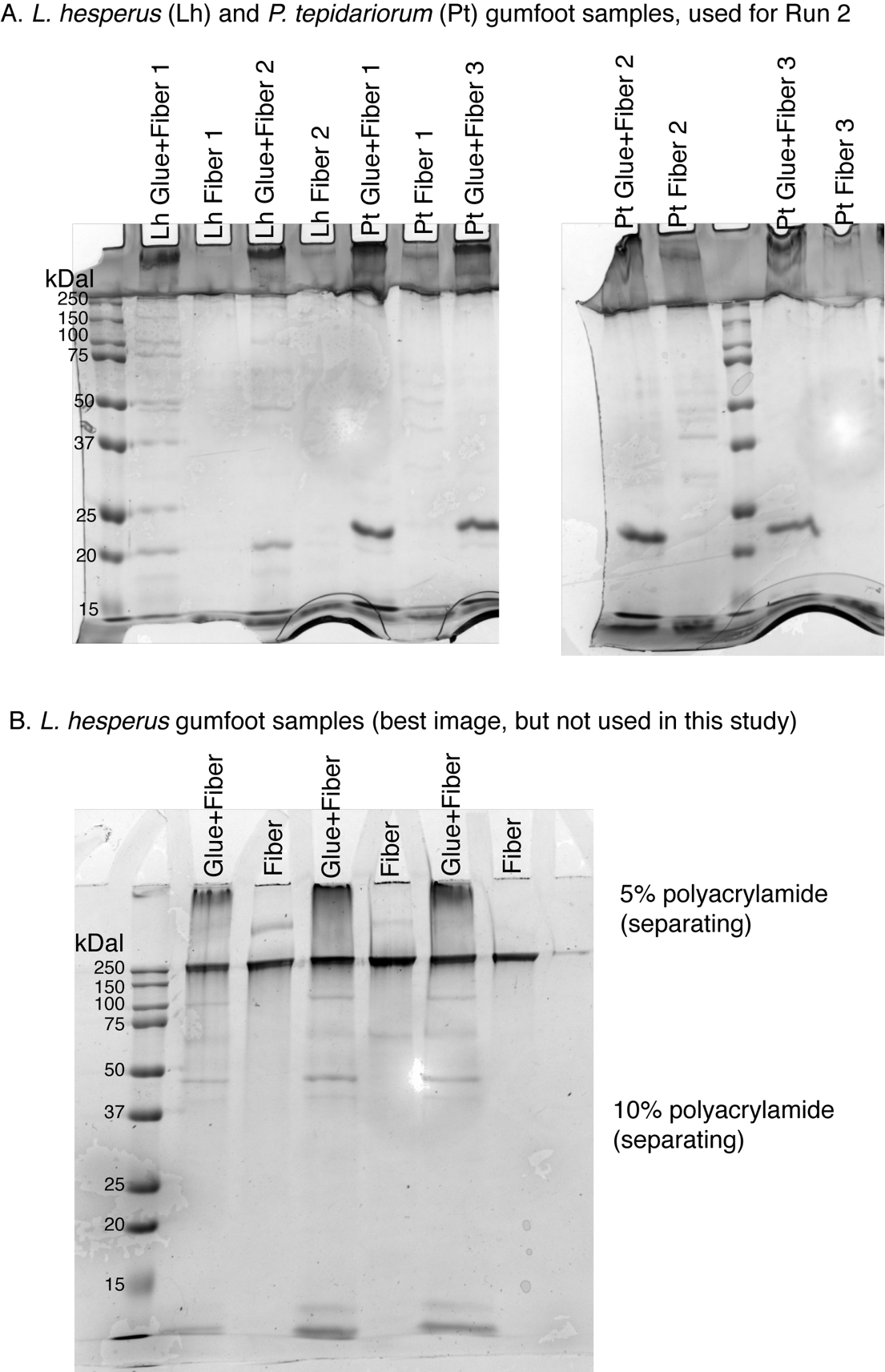
**

Figure S1. Representative SDS-PAGE images for gumfoot silk samples.

**Figure S2**


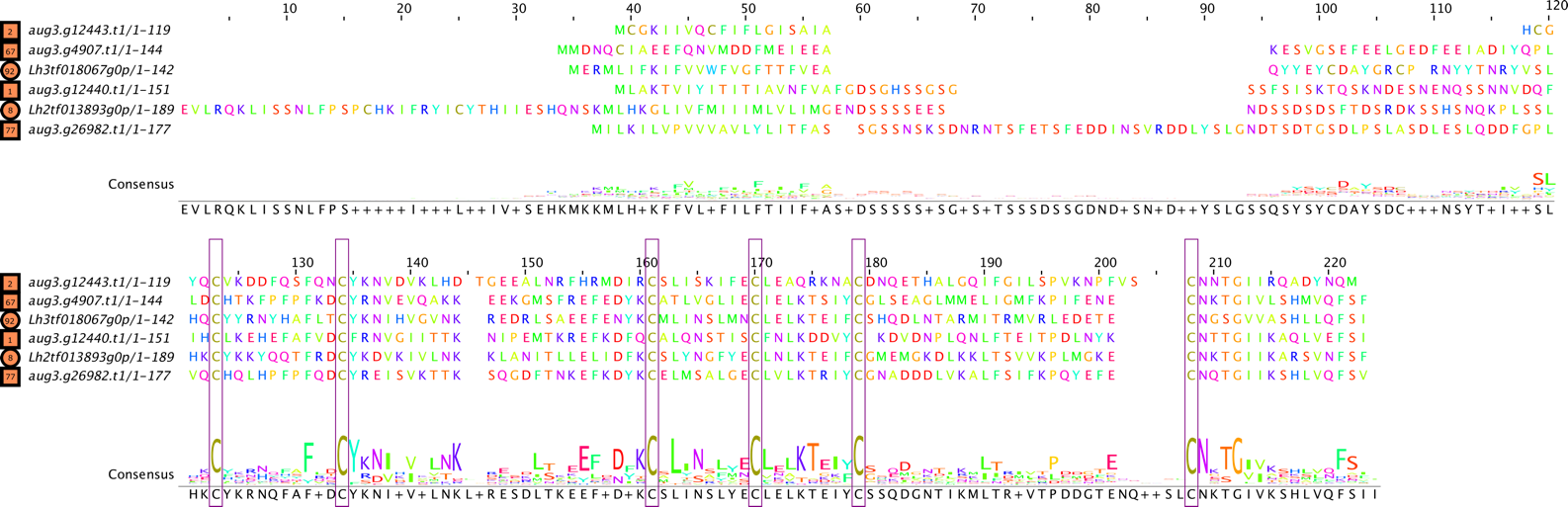


Figure S2. Multiple Alignment of Cysteine Residue Family. A multiple sequence alignment of the *Parasteatoda*-seeded family from Schwager et al (2017) containing 27 genes from the Cysteine Residue family (family id: 029625) was made using MUSCLE on EBI. The family contained multiples genes each from *L. hesperus, L. geometricus, S. grossa,* and *P. tepidaroium,* though none from any non-Theridiidae species*.* Only the six genes in the family that were also identified in gumfoot lines are shown with their protein # as seen in Fig. 7. The consensus logo is from all 27 genes. Since the Schwager et al (2017) families used the original *P. tepidariorum* Augustus annotation and not the newer Dovetail annotation, two genes in the family which are completely identical to Dovetail genes in the gumfoot line are shown with that gene’s protein #. The six highly conserved cysteines in the latter half of the alignment are all highlighted and consensus logo at the bottom demonstrates that this conservation is strongest in the cysteines when compared to the rest of the columns.

**Figure S3**

**AgSp1**

>Argiope trifasciata AgSp1 repeat motif 1 (Stellwagen & Renberg 2019)

GPDGKPLPIEPAGPGTTPGTVT GPDGKPKKFVLPKGAFTTPGSIP GPDGKPIHVQPAGPGTTPGAQT GPDGKINKLVVP TTTTPKGPVGPGGMPLSPYSPQGPGGQPMYPFGPGSPYGPGEQTTTTPIP

Phosphorylated

Glycosylated

Phosphorylated & glycosylated

Lh = Latrodectus hesperus

Pt = Parasteatoda tepidariorum

>Lh3tf000234g12u AgSp1 N-terminal

NRQNSFLSLNMGWLSHAFIAIFLIDVQPIQILGQNAGIAQNDYLVSRESPIQNAGGLGEEVKATGNAGTSNNQAAAVPDAASDSIGGGVPGKRKGPTGDVTLGLLGGLFGGGNTQEKSDCKQHGIVTGLISGIFGGGTNDCGDQHETGEQVTGEYVTGENDTGEKRKKCHKNGLITGFVSGLLGGGAEDDCEDEY

AT GEYVT GEYAT GEYVT GEYVT GEYAT GEYAT GEYAT GEYAT GEYAT GEFAT GE

>Lh3tf000234g1p AgSp1 repetitive

AT GEYAT GEYVT GEYVT GEYAT GEYAT GEYAT GEYAT GEYAT GEFAT GEYAT GEYAT GEYAT GEYAT GEYAT GEYAT GEYVS

SESEETPSFVTIPGP

NGDGRPVQLVPAGPGSTPGTVT DSDGNLIRIILPSGAEVTPGSIK GPNGSSIQIMPAGPGSTPGITTN SKGQIEKILIP QIPAPLSFGSGVNRGSTASYVI

GPGNIHIQIVPGSPGNTPGFVT DAGGTIHRIILPTGGGLTPGSIP GPEGRRIVIQPVSSGTSPGVITD SDGHIATLRIP KLPGGIGVPRDRNRPVFIK

GPQGQPVQIVPGGPGTTPGAVT GPNGNIIRIILPAGANLTPGSIQ GPGSQPIQLQPAGSGKTPGFVTN SDGQIRKVIIP KFNIKNPFGSGTTPAIIS

GNKGQPIVIVPGGPNNIPGAVT GPDGNIYKIILPAGADVTPGSIK GPGSRTIRIEPTGPGTTPGVQTD SDGYIIRIIIP QFPAPGPYISGSTPANIP

GPDGKPIEVVSGGPGTTPGAVT DSNGRIIRIILPSGANVTPGSIR DPKGRTIRIVPAGRGTTPGAITN SDGQIIKVIIP IFPIPNPFVAGTTPMYIS

GPKGQTIQIVPGGPGSTPGAVT DLRGNVIRIILPAGGNVTPGSIE GPGGQPIKLQPAGPGTTPGVVTN SNGKIVKIVFP IFPVPGPFGRRTSSYLN

VPGGHPIQIIPGEPGTTPGTVT NSNGDIIRIILPAGADVTPGFIQ GPGERPIKIVPARPGTTPGIITD DDGKINKIVIP QFPKINPFGPGTTPFTVN

GPNGQPIQILPGGVGTTPGVVT DTDGNVIKIILPAGVDITPGSIE GPGGNPIQLVPAGAGTTPGIVTN SNGKIYKIVVP QYQDPGPYVPG

LTPTRIRGPNRMPNKIPQFPAN KPYDHKPKDQGPFGPGNTPSTIT GPNGRPIKIISAGPGTTPGAVTD PNGSIVKIILP EDSGTSPGNIK

GPGGRPIHIVPAGPGTTPGAKT DKDGNIDTIYLPNYPTGPKGRPG TITI

KSPSGAIQIEPAGPGTTPGTVT GPDGSVIKIFYPIGGQSTPGTIP GPGGKPINIMPAGPGTTPGVKQG PDGSINTVYFP NFPIGLGGIPKEDEESSDVI

GPGGRTIRLIPSGPGINKTHLS QILI

>Lh3tf000234g4u AgSp1 repetitive

GEYET GEYTT GEYTT GEYAT GEYTT GKYSTEGKRKKCHKHGLITGFISGLLGGGSGDDCE

GGYET GEYVT GEYTT GEYVT GEYET GEYAT GEYVT GEYAT GEYVT GEYET GEYAT NEYAT GEYAY GEYAY DEYAT GEYAY GEYAT GEYET

EEYAT GEYAT SEYAS GKYAT GEYAT GEYAS SESEETPTFVMVPGP

NGGTRPVQLVPARPGSTPGTVT NSDGNLIRIILPSGKDVTPGSIK GPDGSFIKIIPAGPGSTPGIIT NSKGQIEQIVMP QIRVSSPSGQGVHNGRTASYVI

GPGNVRIQILPGGHGRTPGVVT GAGGIIHRIILPSGGGRTPGSIP GTGGRRIVIQPVGPGRSPGVIT DSDGQIATVRIP ILPGRISPSRNRNRLVFIK

GPQGQPIQIVPAGAGTTPGAIT GPNGNVIRIILPAGANLTPGSIQ GPGGQPIQLQPAGSHITPGVVT NSDGQIRKVIFP KISINPFGSRDTQTMII

GNKEQRIIIMPGGPNNIPGAVT GPDGNIYKIILPAGADVTPGSIK GPGSRTIRIEPAGPGTTPGVQT NSDGYIIRIIIP QFPAPGPYISGSTPANIP

GPDGKPIEVVPGGPGTTPGAVT DPNGQIIRIILPSGANITPGSIR DPKGRTIRIVPAGRGTTPGAIT NSDGQIIKVIIP IFPIPNPFVAGTTPMYIS

GPKGQPIQIVPGGPGSTPGAVT DSRGNVIRIILPAGGDVTPGSVE GPGGQPIKLQPAGPGTTPGAVT NSDGKIVKIVFP IFPAPGPFGGSTPSYLN

VPGGLPIQIIPGGPGTTPGTVT NSKGDIIRIILPAGA

>Lh3tf000234g3s AgSp1 repetitive

TGEYAT GEYVSSESEETP SFVTIPGP

NGDGRPVQLVPAGPGSTPGTVT DSDGNLIRIILPSGAEVTPGSIK GPNGSSIQIMPAGPGSTPGITT NSKGQIEKILIP QIPAPLSFGSGVNRGSTASYVI

GPGNIHIQIVPGSPGNTPGFVT DAGGTIHRIILPTGGGLTPGSIP GPEGRRIVIQPVSSGTSPGVIT DSDGHIATLRIP KLPGGIGVPRDRNRPVFIK

GPQGQPVQIVPGGPGTTPGAVT GPNGNIIRIILPAGANLTPGSIQ GPGSQPIQLQPAGSGKTPGFVT NSDGQIRKVIIP KFNIQNPFGSGTTPAIIS

GNKGQPIVIVPGGPNNIPGAVT GPDGNIYKIILPAGADVTPGSIK GPGSRTIRIEPTGPGTTPGVQT DSDGYIIRIIIP QFPAPGPYISGSTPANIP

GPDGKPIEVVSGGPGTTPGAVT DSNGRIIRIILPSGANVTPGSIR DPKGRTIRIVPAGRGTTPGAIT NSDGQIIKVIIP IFPIPNPFVAGTTPMYIS

GPKGQTIQIVPGGPGSTPGAVT DLRGNVIRIILPAGGNVTPGSIE GPGGQPIKLQPAGPGTTPGVVT NSNGKIVKIVFP IFPVPGPFGRRTSSYLN

VPGGHPIQIIPGEPGTTPGTVT NSNGDIIRIILPAGADVTPGFIQ GPGERPIKIVPARPGTTPGIIT DDDGKINKIVIP QFPKINPFGPGTTPFTVN

GPNGQPIQILPGGVGTTPGVVT DTDGNVIKIILPAGVDITPGSIE GPGGNPIQLVPAGAGTTPGIVT NSNGKIYKIVVP QYQDPGPYVPGLTPTRIR

GPNRMPNKIPQFPANKPYDHKP KDQGPFGPGNTPSTIT

GPNGRPIKIISAGPGTTPGAVT DPNGSIVIIILPEDSGTSPGNIK GPGGRPIHIVPAGPGTTPGAKT DKDGNIDTIYLP NYPTGPKGRPGTITI

KSPSGAIQIEPAG

>Lh3tf000234g7u AgSp1 repetitive

AGTTPGIVTNSNGKIIKIIVPQYPAPGPYVPGLKPARIR

GPNGIPIQIIPGGPGTTPGAVT GPKGNIIRIILPAGSDTTPGTIK GPDGRSIQIIPAGAGTTPGVVT GPDGQIQKLIIP LFPAPGPAGPGSTPVDVN

GPDGQPIQIVPGGPGSTPGAVT GPNGYIIRIILPAGGDVTPGTIR GPDGQPIQIQPVGPGSTPGVIT NPDGQIKKILVP LFPMPLPFGLVTPSEVK

GPNGQPIQLIPGGPGTTPGTIT DSEGYIVKIILPSGSDATPGTVK GPRGIPIQIIPVGPGSTPGVIT GPDGSIQKIYVP IFPIPSPIFTRTTPTRIK

GPNGQPIQIIPGGPGTTPGAIT DNKGNVIRIILPSGANRTPGSIE GPGGRPIQIRPAGPGTTPGVVT KPDGSIRIIYIP QFPAPEPYDHKPK

>Lh3tf000234g6u AgSp1 repetitive

LLPQGSGITPGSVTLPG GQTVNIIPVGPGTTPGAKTGPD GNIDTLYVPHIP GTQSPRRSTPSQVT

GPGGNPIQLIPSGPGTTPGAVT GPDGKIRFIILPSGSGTTPGSVE GPGGKPIQIIPAGPGTTPGAET GPDGNINKIYIP TGPSPQSPSGSTPSQVT

GPGGRPISLIPSGPGNTPGAVT GPDGRIYYIILPSGSGTTPGSIE GPSGKPIKIIPAGPGTTPGAQT GPDGSINVIYVP RSDQTTPTESSDSKGQGGTTPGSSENIPGTKGPGRTTPVEIT

GVDGHPIKLIPAGPGTSPGAVL GPDGKPFIILLPQGSGKTPGSVT LPGGQTVNIIPVGPGTTPGAKT GPDGNIDTIYVP RKPGTQGPSRSTPSQVT

GPGGNPIQLIPSGPGTTPGAVT GPDGKIKFIVLPSGSGTTPGSVE GPGGHPIKILPAGPGTTPGAET GPDGKIDKIYIP KGPGPQRPGGSTPSQVT

GPEGRPISLIPSGPGITPGAVT GPDGKIYYIVLPSGSGTTPGSIE GPGGKPIKIVPAGPGTTPGAET GPDGKINKIYVP RGDHTTPSENSGTEGGTTPGS

>Lh3tf000234g25u AgSp1 repetitive

TPGSIK GPGGSSIQIIPAGPGSTPGIIT NSKGQIEQIVMP QMPVPLPFGPGVNKGSTASYVI

GPGNIPIQIVPGGPGNTPGVVT DAGGTNQAADIL

>Lh3tf000234g17u AgSp1 repetitive

DTLYLPNYSIVPSGQGTNPI

KSPSGPIQIEPAGPGTTPGTVT GPDGSVIKIFFPIGGQSTPGTIP GPGGKPIKIMPAGPGTTPGIKQG PDGSINTVYFP NFPIGLGGIPKEDEESSDVI

GPGGRTIRLIPSGPGINKTRLS QILI

GVKQG PDGSIDTIYLP NYPIGPGGSPEQTTKSPHGPSTPIEVK

GPDGSPIKLIPAGPGTSPGAVL GPDGKPFIILLPQGSGKTPGSVT LQGGRTVNIIPVGPGTTPGLQTG PDGNIDTIYVP KIPGTQGPQTSTTSEIT

GPSGNPIQLIPSGPGSTPGTIT GSDGRPIKIILPKGSGTTPGTVK GPDGKQIQIIPIGPGSTPGAETG PDGNIRTIYIP RRPGSETPSGSTPSQVT

GPGGRPIQLIPSGPGTTPGVVT GRDGKIRFIILPSGSGTTPGSVE GPGGKPIQIIPAGPGTTPGAETG PDGNINKIYIP TGQNP

>Lh3tf000234g21u AgSp1 repetitive

SPGAVL GPDGKPFIILLPQGSGQTPGTVT LPGGQTINIIPVGPGTTPGAKTG PDGNIETLYVPREPGTQGPSRSTPSQVT

GPGGRPIQLIPSGPGTTPGAVT GPDGKIRFI

>Lh3tf000234g11u AgSp1 repetitive

E GPEGQRIKIIPARPGTTPGAETG PNGNINIIYIP GITSKQEPSNSTPSQIR

GPGGRPIQLIPSGPGTTPGAVT GPDGSIQYIILPFGSGTTPGSIE GPGGNPIKIVPAGPGTTPGARTR PDGSISI

>Lh3tf000234g28u AgSp1 repetitive

YLPNYSIG PSRQGTTLI

KSPSEPIQIEPAGPGTTPGSVT GPDGSVIKILYPMGEQLTPGTIL GPGGKPIKIMPAGPGITPGIKQG

>Lh3tf000234g0u AgSp1 includes C-terminal domain

GSQGPGGSTPSQIT

GPSGKPIQLVPSGPGSTPGVVT GPDGSPIKFILPKGSGSSPGSFE GPNGQPIKVMPVGPGSSPGAKT GPDGSVSIIYVP SQPSGQTTPGSGGPGGSTPSQVT

GPGGNPIQLIPAGPGTTPGAVT GSDGKIRFIILPSGSGTTPGSVE GPKGKPIKIVPAGPGTTPGAET GPDGNINKIYIP TGPQSPSGSTPSQVT

GPGGRPISLIPSGPGNTPGAVT GPDGKIYYIILPSGAGSTPGSVE GPGGKPIKIVPAGPGTTPGAQT GPDGRINIIYIP RGGQTTPKESSGAKGQGGTTPGSSKNVPGPQGPGRTTPVEIT

GVDGHPIKLIPAGPGTSPGAVL GPDGKPFIILLPQGSGITPGSVT LPGGQTVNIIPVGPGTTPGAKT GPDGNIDTLYVP HIPGTQSPRRSTPSQVT

GPGGNPIQLIPSGPGTTPGAVT GPDGKIKFIVLPSGSGTTPGSVE GPEGQRIKIIPARPGTTPGAET GPNGNINIIYIP GITSKQEPSNSTPSQIR

GPGGRPIQLIPSGPGTTPGAVT GPDGSIQYIILPFGSGTTPGSIE GPGGNPIKIVPAGPGTTPGART RPDGSISIIFIP RGPGPQSPTGSTPSQIT

GPGGRPIYLIPSGPGNTPGAVT GPDGTIYYIILPYGSGTTPGTVI GPGGNPIRIIPAGPGTTPGAKT GPDGRISIIYIP QRPGSEQSFNTPASIE

GPQGEPIQIVPGQPGVTPSAVT GPDGTVIKIIYPSGSPVTPGSIP GPGGKPIKIMPAGPGTTPGART GPKGQLIELFLP QYPFGKPSPP

GVTQPNGQPIRIVPGSTPGTVT GPDGNIIEIVLPQDSPVTPGSIQ GPDGRPIKLLPAGPGTTPGAKT DSNGNIIQLILP QYPFGQPQQPQP

DFAGGNGEPIQIVPGTTTAIIT DSDGYISKLILPQDSPVTPGTIT GPDGKPIKIIPAGPGTTPGAKI NPQGQITEITLP KYPFGQPQNNSPPQPLDP GSPGFGISFVFPKYLNGMNL

PGGLLDPSQYPDGINQILTGQPVSYPQLIKFIQPLFPGGQIDPNVIPQDNLYGPNGNFYLPGFQGTFDNLVLVNNGFPDYPNQNPQFGGMLYIPQLLKLVNYIPVSTPNTDFNFPGLNGQLQSFCGYSDTDYSQDYEDMQAPSPYPNSGEALGGAVPGGNQGAGGDMAAPSPNSGTGQCENDAHGTFQKARTALQKVSTADGKQKISKLLTALKSGINDEESVDFNMFFNELSMLFSQVRSESSEVSTDVEFTEVLMEALGAALQVISSAKIVGFETTDVVDVSVYTSYLSEVLF

>Pt MSTRG.24787.11 AgSp1

LIGQFCEHNMGWLSHVIIAIFLIDIHPTKIYAQEVGIAQNDPLLSRETGSNFASSFVNYLKSCGQFTIKEADQFREVLDTLTDAYGDFANLQFLSRTSMMAAAAGFAAAISERTANDVHAGDLMPKSGCVINALKHAFIMSTGVSNPFFISEVRKLIQIFFLTDAAPLDELPEPEYVGDIGGEVAGTNQGPTGDLRAAAPDDLADDDDLGGGEKGKRGGPRGDMEAGIFSAIFGSDGSS

SSYET TEYVT GEYQT GEYVT GEYQT GEYVT GEYET GEYVT GEYET GEYVT GEYAT GEYTT GEYET GEYVT GEYST GEYVT GEYTS GAAASKKCAPKKKGIISGFITGIFGSNGSDDCEEETGEAVSAEYQSYYYYSYIT

GEYYS GVFDS YTYRT DEYIT GEYYT GEFSS TDYYT GEIIT AEQYS GGYGS GEYSS AEIPT FVVLP

GPNGEQRPVQIVIAGPGSTPGTVT NSNGLLVKIVLPSGGDVTPGTIP GSNGRTIQIVPAGPGVTPGIIT GTDGYIIKIVIP SFPHPKPFPHPNKPGRRVIFVT

GPGGRRIQIVPAGPGRTPGTVT DNNGNIIRIILPAGGGISPGSVN GPGGRKIVIIPVGPGTSPGVIT GSDGKIATIKIP PFPRGGRRPHHSTPSYVE

GPGRQLIQIVPGGPGTTPGAIT GPSGNIVRIILPAGADVTPGNIK GPNNRQIYIQPAGKGTTPGVVT GSDGQIATIIIP RFKIRHPFHGGFTPVPIN

GNHGQVILIVPGGPGRTPGFVT AKDGNIVKIILPAGADVTPGSLP GPGGRPIRIEPVRPGTTPGAVT GSDGNIIKILIP QFPMPGPYIEGSTPLTIKL

_PGGRPIAVIPGGPGTTPGVVT DPNGRIIRIILPAGADVTPGSIE GPYGQTIKIQPESPGSTPGAVT NSDGHIITIIIP LLPMPGPFGPGTTPSYVK

GPKGELIQIVPGGPGTTPGVQT GPYGNIIRIILPAGGDVTPGTVK GPGGRTIKIRPAGPGTTPGVRT DSDGYIIEIIIE IFPAPGPFGGSTPAFLRI

_PGRRPIQIIPGGPGTTPGTVT DRKGNIIKIILPGGADVTPGSIE GPDGQPIQIIPVRPGTTPGVIT DPNTHKIIKIII PQFPYKNPFGPGTTPVQIA

GPNGRPIQIIPAGAGTTPGTVT DRHGRIIQIIIPAGSDVTPGSVD GPGGRPIKIVPVGPGSTPGVVT DSSGQIIKIIIP QFPAPYPDIPGATPGMVR

FPNGRPIIIIPGGPGTTPGAIT GPDGTIIRIILPAGGASTPGTIK GPGGRPIQIEPAGPGTTPGVET GKDGHVVKIYLP RFPSPGPAGPGSEPSYIT

GPQGQPIQIIPGGPGTTPGVVT GPYGNIIRIIIPAGGDVTPGDIP GPDGKPIRIEFPGPGTTPGIVT KPGGKIVKIIIP FFPQNLPSGFSTPAEVK

GPDGRPIQIILTGPGSTPGTVT DKEGHIVRILLPNGANKTPGSVK GPGGRPIKIETVQPGSTPGVVT GPDGQIKKIIIP VFPVPGDEPSSESTPKSVPNPDG

GR___PIQLIPGGPGTTPGVIT DSDGHVIRIILPSGADRTPGSID GPGGRPIKIQPLGPGTTPGVVT RPDGSIKIIFVP QFPSSDNNAPGPKDDGPYGPGNTPGSVE

GPDGRPIQIIPAKPGTSPGAVT DASGRIIKIILPVGSGTTPGTVP GPNGHPIHIVVAGPGTTPGSKT GPDGNIIEIVLP NPNRKPEGGEATTPKQVTDKK

___GASISIIPAGPGTTPGAVT DTSGNIIRIILPQNGEKTPGTIK

GPDGRPIKIFPYQKGTTPGVKL GPDGNVIEIHLPKNPFDKP_IIP IKPG

GGGSGPIQIVPEGPGSTPGTVT GPDGSIIKIVFPRGAQSTPGTIP GPGGRPIKIGPAGPGTTPGVEQ NPDGSIKVIYLP SFPIGPGGPGTTKIIPGKPS

GPSG_PIQLVPEGPGSTPGTVT GSDGNPVKIIFPLSAQSTPGTIP GPGGKPIRIGPAGPGTTPGVEQ NPDGSIKVIYLP SFPIGPGTTVTSSGSKGQGGTTPGESGEAPGPSTPSQIT

GPGGKPIQLIPQGPGTTPGTVT RKDGTIIYIILPRGSGTSPGSVE GPGGKPIKILPAGPGTTPGAKT GPDGNIQIIFIP QHPTTRRDGSTPSHIT

GPGGRPIQLIPSRPGQTPGAVT GPDGYIIYIILPTGSGTTPGSVE GPNGHPIRIMPVGPGSTPGAKT GPDGNIDVIYIP QTPKSSTPSQVT

GPGGNPIQLIPQGPGTTPGAVT GPDGTIIYIILPKGSGTTPGSVE GPGGKPIKILPAKPGTTPGAET GPDGTIEIIYIP QYPTTRRDGSTPSQIT

GPGGRPIQLIPRRPGQTPGTVT GPDGYPIYIILPSGSGSTPGSVE GPDGKPIKIMPVGPGTTPGAKT GPDGTIEIIFIP QSESTPTGSTPSQIT

GPGGRPIQLIPGKPGQTPGAVT GPDGYPIYIILPSGSGSTPGSFE GPDGQPIKVVPVGPGTTPGAET RPDGSIKIIFIP QSGSTPSGSTPSQVT

GPGGRPIQLIPSKPGQTPGTVT GPDGYPIYIILPSGSGSTPGSFE GPDGQPIKVVPVGPGTTPGAET RPDGSIKIIFIP QSGSTPSGSTPSQVT

GPGGRPIQLIPLSPGSTPGAVT GPDGSVIYIILPNGSGTTPGSIE GPDGQPIKIMPAGPGTTPGAET GPDGNIRIIYIP SHPKTRPDGSTPSQVT

GPGGRPIQLIPSRPGQTPGAVT GPDGYPIYFILPSGSGSTPGSFE GPDGQPIKVVPVGPGSTPGAKT RPDGSIQIIFIP QSGSTPSGSTPSQVT

GPGGNPIQLIPMRPGSTPGAVT GPDGVIIYIILPEGSGTTPGSVE GPDGKPIKILPAGPGTTPGAET GPDGSIQIIYIP SHPTTRPDGSTPSQVI

GPGGRPIQLIPRRPGQTPGTVT GPDGYPIYIILPSGSGSTPGSVE GPDGKPIRLVPVGPGSTPGAQT GPDGSIQIIFIP QSRSTPSGSTPSQIK

GPGGLPIQLIPQGPGTTPGAVT RPDGSIIYIILPSGSGTTPGSIE GPGGKPIKIVPAGPGTTPGAKT GPDGSIQIIFIP QEQSRPSGTTPSEVK

GPGGRPIYLIPSRPGQTPGAVT GPDGYIIYVILPQGSGTTPGSVE GPGGKPIKLIPVGPGTTPGTKT GPDGVIEIIFIP SSPGGGSTPSQVE

GPGGRPIQLLPLQPGSTPGAVT GPDGSIIYIILPSGSGTTPGSVE GPGGKPIKIVPVGPGTTPGAKT GPDGTIEIIFIS GQPSSPGGSTPSQVE

GPGGRPIYLIPSRPGQTPGAVT GPDGYIIYIILPRGSGTTPGSVE GPGGKPIKLIPVGPGTTPGAKT GPDGVIEIIFIP ASPGGGSTPSEVD

GPGGRPIALIPMQPGSTPGAVT GPDGVIIYIILPPGSGTTPGSVK GPGGKPIKIIPVGPGTTPGTKT GPDGTIEIIFIS GQPSSPGGSTPSQVT

GPDGRIIYLIPSRPGQTPGSVT GPDGYIIYIVLPEGSGTTPGSVE GPDGQPIKVIPVGQGSTPGAKT GPDGSIQIIYIP RRPTTPGSGSTPAPVQ

GANGQPIQIVPGSPGTQPHAVT GSDGFIIEIVIPYGSPVTPGSIE GPNGQPIKIIPAGPGTTPGAKI GPNGRITEIILP VYPFGTTPASPSGGGFTQ

_PDGSPIRIVP___GSTAGFVT GPDGSVIEIVLPADEPITPGSIK GPGGRPIRLVPAGPGTTPGAKT DQNGYIIELILP KYPFGPPLPPSGGLMN

_PNGQPIQIVP___GTTTEIVT GSDGFIVEIILPQDEPVTPGSIK GPDGRPIQILVAGPGTTPGGKT NPKGQLTSLTLP QYPFGLPQNNRPQQPGQPGFGISFVFPKYLNNKHQ

PGGILEPSQFPSGITEIIEGSVSYSSVVTILQSVFPGGTIDPNSIPQSHLQGPDGNFYLPGFTGTMDSLSLLNDGFPDFQQPDDDGQQFGGVFYLPQLLQLIQNIPGTDGNFDFPGLNGQLQSFCKYSNFDYTQGDDYDMQAPSPFPASNDLGGTKPGGNILGGNLKLGSPFFTPRNLGGGTCDTDAAGTLRKARTALQEVSLPQSQQAITTLLRAIQAGVDASDGNVDYNMFFNELSTMVSQVRSGVAGGKPDVGMLQLLFEALIASLEALNSANISGFSAVEVTEDIPIYAAFLQEVFY

>Pt aug3.g13576.t2 AgSp1

MGWLSHVIIAIFLIDIHPTKIYAQEVGIAQNDPLLSRETGSNFASSFVNYLKSCGQFTIKEADQFREVLDTLTDAYGDFANLQFLSRTSMMAAAAGFAAAISERTANDVHAGDLMPKSGCVINALKHAFIMSTGVSNPFFISEVRKLIQIFFLTDAAPLDELPEPEYVGDIGGEVAGTNQGPTGDLRAAAPDDLADDDDLGGGEKGKRGGPRGDMEAGIFSAIFGSDGSSSS

YET

TEYVT GEYQT GEYVT GEYQT GEYVT GEYET GEYVT GEYET GEYVT GEYAT GEYTT GEYET GEYVT GEYAT GEYTT GEYET GEYVT GEYST GEYVT GEYTS GAAASKKCAPKKKGIISGFITGIFGSNGSDDCEEETGEAVSAEYQSYYYYSYIT

GEYYS GVFDS YTYRT DEYIT GEYYT GEFSS TDYYT GEIIT AEQYS GGYGS GEYSS AEIPTFVVLPGPNGEQRPVQIVIAGPGSTPGTVTNSNGLLVKIVLPSGGDVTPGTIPGSNGRTIQIVPAGPGVTPGIITGTDGYIIKIVIPSFPHPKPFPHPNKPGRRVIFVT

GPGGRRIQIVPAGPGRTPGTVT DNNGNIIRIILPAGGGISPGSVN GPGGRKIVIIPVGPGTSPGVITG SDGKIATIKIP PFPRGGRRPHHSTPSYVE

GPGRQLIQIVPGGPGTTPGAIT GPSGNIVRIILPAGADVTPGNIK GPNNRQIYIQPAGKGTTPGVVTG SDGQIATIIIP RFKIRHPFHGGFTPVPIN

GNHGQVILIVPGGPGRTPGFVT AKDGNIVKIILPAGADVTPGSLP GPGGRPIRIEPVRPGTTPGAVTG SDGNIIKILIP QFPMPGPYIEGSTPLTIK

LPGGRPIAVIPGGPGTTPGVVT DPNGRIIRIILPAGADVTPGSIE GPYGQTIKIQPESPGSTPGAVTN SDGHIITIIIP LLPMPGPFGPGTTPSYVK

GPKGELIQIVPGGPGTTPGVQT GPYGNIIRIILPAGGDVTPGTVK GPGGRTIKIRPAGPGTTPGVRTD SDGYIIEIIIE IFPAPGPFGGSTPAFLRI

_PGRRPIQIIPGGPGTTPGTVT DRKGNIIKIILPGGADVTPGSIE GPDGQPIQIIPVRPGTTPGVITD

PNTHKIIKIIIPQFPYKNPF

GPGTTPVQIA

GPNGRPIQIIPAGAGTTPGTVT DRHGRIIQIIIPAGSDVTPGSVD

GPGGRPIKIVPVGPGSTPGVVT DSSGQIIKIIIP QFPAPYPDIPGATPGMVRF PNGRPIIII PGGPGTTPGAIT

GPDGTIIRIILPAGGASTPGTI K GPGGRPIQIEPAGPGTTPGVETG KDGHVVKIYLP RFPSPGPAGPGSEPSYIT

GPQGQPIQIIPGGPGTTPGVVT GPYGNIIRIIIPAGGDVTPGDIP GPDGKPIRIEFPGPGTTPGIVTK PGGKIVKIIIP FFPQNLPSGFSTPAEVK

GPDGRPIQIILTGPGSTPGTVT DKEGHIVRILLPNGANKTPGSVK GPGGRPIKIETVQPGSTPGVVTG PDGQIKKIIIP VFPVPGDEPSSESTPKSVPN

PDGGRPIQLIP GGPGTTPGVIT

DSDGHVIRIILPSGADRTPGSID GPGGRPIKIQPLGPGTTPGVVTR PDGSIKIIFVP QFPSSDNNAPGPKDDGPYGPGNTPGSVE

GPDGRPIQIIPAKPGTSPGAVT DASGRIIKIILPVGSGTTPGTVP GPNGHPIHIVVAGPGTTPGSKTG PDGNIIEIVLP NPNRKPEGGEATTP

KQVTDKKGASISIIPA

GPGTTPGAVT DTSGNIIRIILPQNGEKTPGTIK GPDGRPIKIFPYQKGTTPGVKLG PDGNVIEIHLP KNPFDKPIIPIKPGGGGSGPIQIVPEGPGSTPGTVT

G PDGSIIKIVFP RGAQSTPGTIP

GPGGRPIKIGPAGPGTTPGVEQN PDGSIKVIYLP SFPI

GPGGPGTTKIIPGKPSGPSGPIQLVPEGPGSTPGTVTGSDGNPVKIIFPLSAQSTPGTIP

GPGGKPIRIGPAGPGTTPGVEQN PDGSIKVIYLP SFPIGPGTTVTSSGSKG

QGGTTPGESGEAPGPSTPSQI TGPGGKPIQLI PQGPGTTPGTV TRK

DGTIIYIILPRGSGTSPGSVE GPGGKPIKILPAGPGTTPGAKTG PDGNIQIIFIP QHPTTRRDGSTPSHIT

GPGGRPIQLIPSRPGQTPGAV TGPDGYIIYIILPTGSGTTPGSVE GPNGHPIRIMPVGPGSTPGAKTG PDGNIDVIYIP QTPKSSTPSQVT

GPGGNPIQLIPQGPGTTPGAV TGPDGTIIYIILPKGSGTTPGSVE GPGGKPIKILPAKPGTTPGAETG PDGTIEIIYIP QYPTTRRDGSTPSQIT

GPGGRPIQLIPRRPGQTPGTV TGPDGYPIYIILPSGSGSTPGSVE GPDGKPIKIMPVGPGTTPGAKTG PDGTIEIIFIP QSESTPTGSTPSQIT

GPGGRPIQLIPQGPGSTPGAV TGPDGVIIYIILPRGSGTTPGSVE GPDGQPIKIMPAGPGTTPGAETG PDGNIRIIYIP SHPRTRPDGSTPSQVT

GPGGRPIQLIPSRPGQTPGTV TGPDGYIIYIILPSGSGSTPGSVE GPDGRPIKIVPAGPGTTPGAKTG PDGSIQIIYIP QPKPSSTPSQIT

GPGGRPIQLIPLSPGSTPGAV TGPDGVIIYIILPRGSGTTPGSVE GPDGQPIKIMPAGPGTTPGAETG PDGSIRIIYIP SHPRTRPDGSTPSHVT

GPGGRPIQLIPGKPGQTPGAV TGPDGYPIYIILPSGSGSTPGSFE GPDGQPIKVVPVGPGTTPGAETR PDGSIKIIFIP

QSGSTPSGSTPSQVTAGPGTTPGVKTRPDGSIKIIYIPQSQGTTPSGTTVTSSGSKGQGGTTPSGSSNAPGPSTPSGSTPSQVT

GPGGSPIQLIPLSPGSTPGAV TGPDGR

_PGGRPIQLIPGKPGQTPGAV TGPDGYPIYIILPSGSGSTPGSFE GPDGQPIKVVPVGPGSTPGAETR PDGSIKIIFIP QSGSTPSGSTPSQVT

GPGGRPIQLIPLSPGSTPGAV TGPDGSIIYIILPNGSGTTPGSVE GPDGQPIKIMPAGPGTTPGAETG PDGSIRIIYIP SHPRTRPDGSTPSQVT

GPGGRPIQLIPSKPGQTPGTV TGPDGYPIYIILPSVKLN PDGSI DTIYLPTFPIGPGTTVTSSGSKGQGGTTPGESGEAPGPSTPSQIT

GPGGRPIQLIPLSPGSTPGAV

---------TGPDGRSTPGAV TGPDGRIIYIILPNGSGTTPGSVE GPDGQPIKIMPAGKGTTPGAETG PDGSIRIIYIP SHPRTRPDGSTPSQVT

GPGGRPIQLIPSRPGQTPGTV TGPDGYPIYIILPSGSGSTPGSFE GPDGQPIKVVPVGPGTTPGAETR PDGSIKIIFIP QSGSTPSGSTPSQVT

GPGGRPIQLIPLSPGSTPGAV TGPDGSVIYIILPNGSGTTPGSIE GPDGQPIKIMPAGPGTTPGAETG PDGNIRIIYIP SHPKTRPDGSTPSQVT

GPGGRPIQLIPSRPGQTPGTV TGPDGYPIYIILPSGAGSTPGSFE GPDGQPIKVIPAGPGTTPGVKTR PDGSIKIIFIP QSHGTTPSGTTVTSS

GSKGQGGTTPGGSSNAPGPSPSDQSTPSQPIVVTLSPIS

GPGGEPIQLIPGSGGSTPGVK TGPDGQPITIVLPEGAGSTPGTIE GPNGEPIKVIPIQPGTTPGVKTG PDGSISIIYVP QSSSTPTDRKTPRQPIVVTLSPIS

GPGGEPIQLIPGSGGRVKTGV

IPIQPGTTPGVK TGPDGSISIIYVPQTSSTPQ GPDGEPITSSGVKSQGGTTPGGSDIAPGPSTPSQVT

GPGGRPIQLIPLRPGSTPGTV TGSDGYPIYIILPSGSGTTPGSVE GPDGTTPGAETGPDGSIRIIYIPSHPRTRRDGSTPSQVT

GPGGRPIQLIPSRPGQTPGAV TGPDGYPIYFILPSGSGSTPGSFE GPDGQPIKVVPVGPGSTPGAKTR

PDGSGSTPGSFEGPDGQPIKVVPVGPGSTPGAKTRPDGSIQIIFIPQSGSTPSGSTPSQVT

GPGGNPIQLIPMRPGSTPGAV TGPDGVIIYIILPEGSGTTPGSVE GPDGKPIKILPAGPGTTPGAETG PDGSIQIIYIP SHPTTRPDGSTPSQVI

GPGGRPIQLIPRRPGQTPGTV TGPDGYPIYIILPSGSGSTPGSVE GPDGKPIRLVPVGPGSTPGAQTG PDGSIQIIFIP QSRSTPSGSTPSQIK

GPGGLPIQLIPQGPGTTPGAV TRPDGSIIYIILPSGSGTTPGSIE GPGGKPIKIVPAGPGTTPGAKTG PDGSIQIIFIP QEQSRPSGTTPSEVK

GPGGRPIYLIPSRPGQTPGAV TGPDGYIIYVILPQGSGTTPGSVE GPGGKPIKLIPVGPGTTPGTKTG PDGVIEIIFIP SSPGGGSTPSQVE

GPGGRPIQLLPLQPGSTPGAV TGPDGSIIYIILPSGSGTTPGSVE GPGGKPIKIVPVGPGTTPGAKTG PDGTIEIIFIS GQPSSPGGSTPSQVE

GPGGRPIYLIPSRPGQTPGAV TGPDGYIIYIILPRGSGTTPGSVE

GPGSGTTPGSVEGPDGQPIKVIPVGQGSTPGAKTGPDGSIQIIYIPRRPTTPGSGSTPAPVQGANGQPIQIVPGSPGTQPHAVTGSDGFIIEIVIPYGSPVTPGSIE

GPNGQPIKIIPAGPGTTPGAKIG PNGRITEIILP VYPFGTTPASPSGGGFTQPDGSPIRIVPGSTAGFVT

GPDGSVIEIVLPADEPITPGSIK GPGGRPIRLVPAGPGTTPGAK

TDQNGYIIELILPKYPFGPPLPPSGGLMNPNGQPIQIVPGTTTEIVTGSDGFIVEIILPQDEPVTP

GSIKGPDGRPIQILVAGPGTTPGGKTNPKGQLTSLTLPQYPFGLPQNNRPQQPGQPGFGISFVFPKYLNNKHQPGGILEPSQFPSGITEIIEGSVSYSSVVTILQSVFPGGTIDPNSIPQSHLQGPDGNFYLPGFTGTMDSLSLLNDGFPDFQQPDDDGQQFGGVFYLPQLLQLIQNIPGTDGNFDFPGLNGQLQSFCKYSNFDYTQGDDYDMQAPSPFPASNDLGGTKPGGNILGGNLKLGSPFFTPRNLGGGT

CDTDAAGTLRKARTALQEVSLPQSQQAITTLLRAIQAGVDASDGNVDYNMFFNELSTMVSQVRSGVAGGKPDVGMLQLLFEALIASLEALNSANISGFSAVEVTEDIPIYAAFLQEVFY

**AgSp2**

*>Argiope trifaciata* AgSp2 repeat motif (Stellwagen & Renberg 2019)

QPGSQPIQVKPAQPGTTPGVVTGPDQKPSQVIVPPGGGSTPGTLPGPGGKPVQVEPAKPGTTPGAITGPDRQVSKIILPTGPG

>Lh3tf002378g0u AgSp2

TYSKTVFLIPNMGCSAVAILALCLISINVQTFAQEDTDPFDFNETNEEFGKLFVNNLIESGVFGQTDDKDFNAVTESLLNAIKMLTKGQNAPASTKKTFMMAFASSLAELIVQESDNALSLVEKTRAVTDAMRKAYMQTSGNPNEALIQKVDFLVGIFLDVQQSAEDYEYD

TIVFEDEMPLPLEIVGETLPNIDP

SPGHPDIVMESPPEVTPGIVM GPGNDIKEIVLPQSIESHMDEPETYI GEVDKMIKFEQDAPSGPN APEEQIQQIMLP GMSQPGEDNDEGDAQKIIIPDSS

PLEEIPIPSAPVPIPSLNPFN VAPFPIPNLEPIDAALTPILDPFMPL ITPAISPPKLEEPPKSDD SENMQIQQIMMD

TDGEPGEPSPEETKIVQEGSNSEPIPEEIMPLLDLIPEMNEPIEGDTIIEQKGTDEG SPGMQIQQVMPP KVSPESSGEQVINL

PEPQISPTKDLIGPFGMPGQVDILNQPQISPFGGFGYPYPMIPGSMPENVGDQQPFSNPFLQNLMKKFNPTYLFGSPYQGIGL

NAIEQTFNVVLRNGETYIPAIMSAIIESKKTAPQTLDLRVLVNKLSPLFSDMTVENFYLSRTEIFIELILEALISSLEIIRMADDSCINKVSAITSPYKYVNAFNDILF

>Pt MSTRG.29636.6 AgSp2 includes N-terminal domain

ITVYKGEILRSCSYTLIRCIMMPNIGWSALAIVAICIVSFGQSDARMAFSDVMGFDEMNDQFAQLFVDNIIESGVFGKSEDKDFTDIVENLMQSIAMFTKLHTEPPDQKKTLMTAFASELAELIVAECDDDAMSAVEKTKSVTDALRHAYVSLGFRPNETFIKEVDFLVGVFLNVAAQDAEMDYD

FQMAQRETRREGFVL

GPGGLPPPQNRYTQYGQETFNV PQVQPPP

PPQLTKGVKAAGMNPAPVKTGLAQ

PPQPAKRVQPVKPVKPTEPKLVQPAKPAQPVK

PPQPVKPVE

PPQPVKPVE

PPQPVQPVQPVHPAQPVQPVE

PPQPVQPVKKPKHAPR

PPLSPHLPHRMTVYKMRGNTYNGQ

DLGEYKPNIEYAQRGTTPGIVF GADNRILKIILPEGAEYVHPQTVIGQVNELVAVLQEGGGKKHGS- GPDYQIQQVMLP GLFGIGKGGPKEVTIMGP

REQIIRIMRQKRSKGITPGIVT SSDKTIQTIILPKKNPANQYFQVK

GPDGELVDIFLGASGSTPKVIR DNLGRISKVILPEFTEPGTITLQP PNLQTIHISPEDIKNLFGSD ISAHQIQQVLMQ RTETGPGQQTISIDSDSSDSSAP

EPVVPAAKPVEPVPEPVEPAPAPLPVPQPAKPLPTPEPSISESSSESSSKETSINIIQEGGGSSPGTSSGPSNQIQQIMLDSGSGSEPEAGHEQTISIGSKSTSSVP

KPVESTPKNKQSISIHGKDTKPSGSQPAEPTPIPAPLPVE

KPVEPAPKPVEPAPTPSPLPVEPTPALEPVKPLPTPEPER---SSESSTKQTSINIMQEGGGSSPDTSSGPSNQIQQVMLDSGSGSEPEATHEQTISIGSKSTSSVP

KPVQSTPKNPKKKQSISIHEKGTKSSGSKPVEPAPIPAPLPAE

KPVEPVPKPIEPAPTPSPLPVEPTPALEPVTPLPTPEPEP---SSESSTKQTSINIMQEGGGSSPDTSSGPSNQIQQVMLDSGSGSEPEATHEQTISIGSKTTSSVP

QPIESTPKKKQSISIHEKDTGSQPVEPTPIPAPEIPQPLTPVQPETVSYTTGAL-----GGGLIPPVNQGSLS

PVPQPLEPSPIPEPIPTPVQPLIEPVSSPEDSSSSSEE--------GSINIVQD-----GGGSDRDDSSSPTNQIQQIMMSDSEPGQSKNQIISIDDQTATPTNNLIKES

PLPEQDITPVPEPVPEPLPKPHHHHHHHHHHHKRH GINIVQR------GGG---HKHRNSRTQIQQVMLPGDSDSESDIDYEKPRQQIISLHMGKGPQTAPIPDIQQ LLLLLYQLYLHLT

>Pt MSTRG.29636.1 AgSp2 includes N-terminal

IPVYKEGILSFCFNTLNRCTGLPNMGWSALAFITICILSIGQSHAQRNQDVMGFDEMSEQFAQLFVDNIIASGVFGKSEDKDFTEIIENLMRAMAMFTKLHKEPPSQKKTLMTAFASELAELIVAECDDDALSVVEKTRAVTDALRHAYRESGFRPNEEFVHEVQMLVGVFLNVAEENADREYEFEMTRKRSPFGNIPQKPLGAGFNFQGGQRTAPQQQPAKILHEQHTQGGYRPGVRDTKFTEETKRTEET

YTQYGQETFNVPQVQPPP

PPQLTKGVKAAGMNPAPVKTGLAQ

PPQPAKRVQPVKPVKPTEPKLVQPAKPAQPVK

PPQPVKPVE

PPQPVKPVE

PPQPVQPVQPVHPAQPVQPVE

PPQPVQPVKKPKHAPR

PPLSPHLPHRMTVYKMRGNTYNGQ

DLGEYKPNIEYAQRGTTPGIVF GADNRILKIILPEGAEYVHPQTVIGQVNELVAVLQEGGGKKHGS**-** GPDYQIQQVMLP GLFGIGKGGPKEVTIMGP

REQIIRIMRQKRSKGITPGIVT SSDKTIQTIILPKKNPANQYFQVK

GPDGELVDIFLGASGSTPKVIR DNLGRISKVILPEFTEPGTITLQP PNLQTIHISPEDIKNLFGSD ISAHQIQQVLMQ RTETGPGQQTISIDSDSSDSSAP

EPVVPAAKPVEPVPEPVEPAPAPLPVPQPAKPLPTPEPSISESSSESSSKETSINIIQEGGGSSPGTSSGPSNQIQQIMLDSGSGSEPEAGHEQTISIGSKSTSSVP

KPVESTPKNKQSISIHGKDTKPSGSQPAEPTPIPAPLPVE

KPVEPAPKPVEPAPTPSPLPVEPTPALEPVKPLPTPEPER---SSESSTKQTSINIMQEGGGSSPDTSSGPSNQIQQVMLDSGSGSEPEATHEQTISIGSKSTSSVP

KPVQSTPKNPKKKQSISIHEKGTKSSGSKPVEPAPIPAPLPAE

KPVEPVPKPIEPAPTPSPLPVEPTPALEPVTPLPTPEPEP---SSESSTKQTSINIMQEGGGSSPDTSSGPSNQIQQVMLDSGSGSEPEATHEQTISIGSKTTSSVP

QPIESTPKKKQSISIHEKDTGSQPVEPTPIPAPEIPQPLTPVQPETVSYTTGAL-----GGGLIPPVNQGSLS

PVPQPLEPSPIPEPIPTPVQPLIEPVSSPEDSSSSSEE--------GSINIVQD-----GGGSDRDDSSSPTNQIQQIMMSDSEPGQSKNQIISIDDQTATPTNNLIKES

PLPEQDITPVPEPVPEPLPKPHHHHHHHHHHHKRH-----------GINIVQR------GGGHKHRNSRTQIQQVMLPGDSDSESDIDYEKPRQQIISLHMGKGPQTAPIPDIQQ

>Pt MSTRG.29636.10 AgSp2 with C-terminal Domain (could not be distinguished from MSTRG.29636.11 in the mass spec)

ARGASINSTTNKESSTKDTSINIMQKGGSSSPGPSSAPKNQIQQIMLSEDS

KPSKEESKPEKPKEPDQIVKPAEPVPELSPEP---------KKKEKKPSKKTSINIMQKG-SDDKPGEKAPKNQIQQ--------VMQTEDSKSKPSEQKIDISSDSTPD

SPVPEPTPVPVPELEPLPAPQPEP

LPAPQPEPLPVPQPEPLPAPQPEPLPAPKPLESSPES-----KKTTEE----KSINIVQEGTGSQPGGKAPKNQIQQIMVSSSSKPSKEVKSKSEQPSEPIPLP

QPAIPSIAPEPAQVEPIPTPKLDEPPP-----------------KDKTSSKKTSINIMQERTGSKPGDKAPKNQIQQVMISSDSKPAKDAEPTPLPQPAAPQPVEQVPEP

ALTTGVKATGLVPPTQEGSAAPPPLRSGDRLIAPDEESYSRFRYPQRHMGYQIEPQMEYLPVQISPGYENMGYSPYQGLGLS

QIENVFNAILRDSKSTIPSIIRILLKAKGTAPQTIDLRVIVNELQSIFSDLQMDHFYLSRPELFMELIMQTLISAFEIIRVADYQCIRNPPALGNTFKYINAFNDILF

**AgSp-like**

>Pt MSTRG.5701.t1 AgSp-like

MQNVNLNFNRRKQGFDPMNEYFNFETNDYPDENYPVKNNPYRQNHFPGGNHRNGNNRNGRNNYPGRNKQNGHDNYNQMTYRKMNGPNGNNFPGNPRRNKPNGRNPNGPGKNKPYGQILHPGKNNRNGMKPHGKNNVDIEKIAHVETYYPVGSDPRSVS

MVINEPVVIRKQIPVKEEVSRQFPEDPYEFDEMNVKFRDIFVTNILSDPNFGTRARTDFKQVTHVMLKAISLLQTAETDPSQKDVLMVSFAATISELIIVECDDSVTLDRKVQIVTRALRNAYLETTGKPNEPLIREVKLIIGILLE

EEEHPIKRLLPNVFPKRGPEETLIPRRGTQIFQNFPTNIIQIQAFLPGFQEQYPLHHHYVKKFPTLTHFINKGKAPPEKHKQCLYELIEFILEQCRRHYEEKNPGVRIPLYTRKPTGFDIFIQSPTTRAPPTGFEIYYSRETTTTLAPIDIEFPPDTTPEERRYVIFFLRRHPYRGQKKNYYKYIKYIIRKYRKKRRKSTTTTTTQQTTKRQVEITLTQPSTQTTTANTVEITLHEITTTKR

PIQINITQIETTAQP

GPGGNPIQLIPMRPGSTPGAV TGPDGVIIYIILPEGSGTTPGSVE GPDGKPIKILPAGPGTTPGAETG PDGSIQIIYIP SHPTTRPDGSTPSQVI

TTVTMKDKTRYCNNQTEPTAV TNSDGQIILLILPQGPGGTPRPIT GPNKQEIKFIESRPGTTAGTVTN SEGDIVEIILP RHSYTDRSTPRPIT

GPNRQTIKFIESRPGTTPGTV TNSKGDIIQIILPRHSYTETTLND RSLIFEKPATGLLDFMRQVVIHG TTPHMIK

GPNGGEIKIIYCKREKATKITTTTMKPFSIAVTERTTVKKPFSIAITTTMKPFSIAVTETTKKLPFSIITEQTTTITTTKT

PLPYCDNSSEPKAV TNADGQIILLILPQRPGTTPRPIL GPNRQMIEFVESQAGMTPGTVTN SKGDIIKIILP RPSFTGSTPRTILFDRSLIFEKLTSRTDFMREVIIH

GTTPHVIKGYGDQEIKIVYCRREKVPKKRIQSTTSVPPIEISVTQSSTTPKPLKITLT

EQLTTRKPIDYCDNRKEPYAV TNSKGQIVLLVLPQRPGTTPGPII GPNKKEIKFVELLPGMTPGAITN SEGQVIEIILP KPSYTGTTPILFDRPLVFEKASTKR

GGHRNVIIHGTTPHLIKSQDGQKIRIIYCKRERTKKPLTTTVLRTTTVSTIATTSNIPLYLTTLPTSTNTMEIVIRGTTPRVII

GPGNRKISIIYCAPESEPQVI RDNEGHIILLILPQHPGSTPAPII GPNGQLITFIESFPGSTPGTVTN GNGDIIEIILP ALSTTTPSTTL RSLDYVRQIVIDGTTPHMVI

GPDGREIRVIYCNPDTEPQAV TDSDGHIILLLLPQKTSTTPMKVT GSQIGVVPKRPDYKIIYG

SPD------------LEPQAV MGTNNEIMLLLLPQHPGEIPERIK CPNGKMVRLREEKPGLKPGYRTN AHGQIIEIIIP

GRSGAVKQGYPGFVVVNPRGE KETHYQVDLGDRPFGRPQYEFLYPSQREQHALLSLR

QFENIFAVIMKTGTERVKELISAILKSKGHSPGTFNLRVFVRELSAIFGDMKFENYFLNYPQLFIELLMEALIAALEIIRSLELQCLENHSETSVAEMTKYVDVLKNVLV

>Pt aug3.g8794.t1 AgSp-like

MSTQRFGRGEKAFRPSRESIRYGEFDHALLKEEERKRIMPIYKDRYYLADEAIEIQRRCAESCMDMIGNPLYRNSFPDKKKLENMYKDIYMYEILVELKESEHQISSPDEAKAPTETTMELETPKPEIMETTPEITVDPVPVETSNKTDPDTKEQKEPEPELKEPPTKKRKKRKKKTPTNEPGKENDSTTTAIQQEAPTPASATDRTSPETDGQSENLHPCRKLKILIRGLKTDVEIPEIEKSLTDFGLRPMKIEQMRKRRGQELKLIPLYLVILTDVPEHREILKVDRLLDTPVRVERFRGGRYEIQCFRCQGFGHTQRNCTATPACMKCAGNVFKLLSFHTFHSTILRLKLEASTSKMWLLFILGFAVLTDTGTGTLFGGGSCNGIVGTVFGCGGKASGSAS

GEYTT GEYTT GSYIT GSYVT GEYTT GEYTT GEYTT GEYVT GEQTS GEYTS REYVT GEYVT GEQKS GEYTT GEYTT GEYTT GERAS GEYTT GEYTT GEYTT GERAS

GEYTT GEYTT GEYTT GERAS GEYTT GEYTT GEYTT GEQAS GEYAT GEYTT GEYTT GERKS GEYVS GEYPS GEYDS GDLLT GEYPS YEYIT GEIPS GKYPS GQYPS

GGNYCFDANFYVLPGGQFPGGPGGQNPSNPLQRYILRFMFPSGQYPNGFFLAAEIPSLQLDSVGSIQVFPLGTFPFDKLGPLAKLPLNQLGNLPLGQLAGKLPGLSPSD

IPDGLANVPLRKLITLHLGPLGNIPLASIPSDAPGADTPLSSLGNTPLGQLPTKLGNFPQFQFILPGQPGQQPPNGKQPAKNFITLHLGPLGNIPLDQ

IPDGLANIPLSQLGKVPANKFKVQFNLPLPLGSLPSGVGKLPLKFIGRLHLGPFGNIPLKDLPDSVANMPLDQLSKLPLKKLPGLLKGLPPSQFSSVPRSSGD

IPDQLSPLTVGDIGSLNFEPLGHIPLKK

IPDTLKNVPLKELDSTPISSLPD

ELGSTPLSSFASISFGPLGNFPWSSLPDEIASKPVGQLASTPISSIPKPRDRPGKARLPFFP

GGPGPVNYPGFLPGGNGPIVDPGFNPFPGYYPGNGKAPGTVQLPGMIYPGGRRP

GQYPAGFPGDVPASSVGSISVFPIGSFPMSKLGPLGSLPIGQVKDLPLGKVPGLLPGASSSGVPDQLASVPLRKIIRFKLGPLGVIPFSSVPSSLDSQPLKVLFKTPLNKIPGLLGNNPKLKGPDGKLPDGSTPLKDLGSIEMPPFGKIPLNNVPDNVAPTPLKSLEKTPVNKLPDKLGKSPPYGKLPDQVGSVPTSKLGRLDLGPFGKLPLRDVPDGLGKLPLGKLAHLPLKQLP

GLLKSLPSSKYSSLPRSSAQ

IPDTLSPLSVGDVGTLSFGPLGRIPLKKLPDKLQDVPLKDFGSTPLRSLPDKMGSTPLSSIGAISAGPFGSIPLSEVPGQP

GGRQPGGSPGQPGRPGGRQPGGSPGQPGGRQPGGFPGQPGGRQPGGFPLLPRQPGQHGKPPKYPSL

GEYPGGQLPDALSSAPLSDVGTVSVFPL

GSFPFSSLGPLASLPLNQLKNIPLGQLPGKIPGINPSD

IPDELKSTPLNKLVRFILGPLGSLPLSS

IPDSEGSEPLSSLGKTPLGKVPDKFPGIPKLPKGGKTPDKSTPLSSVGSIDIVPF

GSIPLSSVPDSIASVPLSSLLKLPAKKLPDLLGKKPPYGHLPDSLGKVPVKNLGRITFPIGSIPLKD

IPKTLYSKPLSQLVSLPLGKLPDILKTLPSSERPSDFPSSSSQ

IPDNLKSVPVKDVASISFGPL

GSAPLTHVPDEIKSKPLG

TFDVPLSSLPDVLSSIPVGKLGKIDEGPTGSEPLSSVPDSVASKPIGKLGTLPLGSVPTKPKGGLPDFTSPLTIQFLFPNGKPDLSKLGNLLSPSTPNS

IPDLTDTINNLLSQLDNESPGSQGSDAPDLEKLGKELLD

IPDQLSNLQLPQDEPKLGKLGTRLILILIKLGTYKLPVGSQPDLGPVAQQLIPILGKLAGLLLPQIIIKLQFVPQSDMPSLPNVPDFSSNPTQAFTLPLRILLKIFGSLGGIPLPQSTPSGSIELSGYSTPEGISFTSSADDTLNYPILQFIFMIGRPGQ

SQQPSTPQFGGLTLDEGNLGSVLSPDLPKTVPELEDLANSLISQLDGQSQPSKLPQLVQLGKILIQIIDQEGQLS

LPDDKQQLGNLGIRFIIIICKLGAYRQNPSPSDFGQQGVKLIRVLGKMAALSLPQMLTDLGTLQIQGQLDLPSG

VPDVGQGAANTYSFPLYILLKIFGNMGSIQLPPSNGSGGFDLSGYSLPNGVSNFPSSSQQILNDTPSQLFYVLGSDTPGAGGSFSYQMYPEGGSPGNGGGGGGSPGGQTTYNIDLSNLSPEQLKNLDPQSLQKLKDYVKTLSPSQLQNL

DLSNIPGLNLGGGNLQVGGNGGNGGGNGGNGGGNGGNGGGNGGYGGGNGGNGGTTTILGGGGGGGGGGNGGGPTTIDLGTLNLNDLTESQLQQFPPQIRQKLRKLNLKFYKLKLRLSNNTATAKWRAFFQFLVSI

>Pt aug3.g5696.t1 AgSp-like

MGWFFVTLSILWVTIVVFEFLFCISYARNHEDQQNKNSKAYSEKQAKFVNAFTKKMSNSGIFGDKFLINFMPITMGLLNAAGKLTSHIDDPGQLDQRSKMSFATSMASLFRKYGTIDIDEISLALTNALKYSFRKINVIANNHISKELSTLVKFYHNFNSEKPVKFVNTFTKKMLHSGIFRESLLINYMPITMKLLRASEKLTSHLEEPALLDQDTKISFAKSVAWLFGKYCNASSTDETSLALTKSLRSSFKKI

NVTPNEDLFKELSTLVNSNLDLNSKKQKKFVNQFTKKMLHSGIFGDNLWINFIPISSELISVAKKLISGLEEPDMLDRDSKVDFASSIASLLGKYCSANNKEKINLELTNALRYSFISADVTPNEDLINELKMLV

EFYFNFNKAADVLHLAEQRLMQSENPNEQGLATRNSGPLFGNADNGNFISSALLQKSVNYKNKDDFLENYDNGNPGNNYDENTDNIIPVQSDSSHENHDTGTGKSFVNPIDNNPVSESSKHKFNFKDISTDSFNKYNSLSDIQNLILDGQFFENELGNEQEDYDVPSFSNVGNMNLPDTFTSKNTLYNENSMTKIYENKNPFNEYFGKKKLVYGVSPPLTFDKYDYNNTSYNFFQDDTSYGLNFPLETMNPFDIFAAKNIREDNEILSEEKFQNNILNDDYFDAVNLDYDNPAPLIVENATFNHNNSKYSFLYSKNVNEINFPGDTVE

NVTPFDLFAAKNLLDHKIFPQEELDDTNSEDEYFDPENIDYDNAPPVTIEKSIFNNGGAILPDTKDLHVPIATVK

NVTPFDLFAAKNLLDHTIFPQEEFDDTNSEGGYFAPKNIDYDNAPPVTIEKSIFNNGGAILPDTKDLHVPIETDLHVPIETVK

NVTPFDLFAAKNLLDHKIFPHKEFDDTNSENEYFAPKNIAYDNASPVTIEKSIFNNGGAILPDTKDLHLPIETVK

SVTPFDLFAAKNLPDHKIFPQEEFDDTNSEDEYFAPKNTDYDNASPVTTEKSILNNVGSILTNTKDLHVPIEKGE

NLTPFDLFAAKYLLDDKVFPKEEFDDTKLEDEYFAPKNIDYDNASPVTIEKSIFNNGGAILPDTKDLHVPIE

AEFDDTNNEDDYFAPENIDYDSTPPVTIEKNIFNNGGPILPDTKDLHVPIETVE

KVTPFDLFAAKNFLDDEIFPKEEFDDTKSKDEYLVPANLDYDNASFVSIEKNIFNNGGSNHNLLPDEYSKKLRFPNETVK

NATSFDLFGAKNLLDDKIFPKGEFDDTELEDGYLVSENLDYDNFPPPTVEYNDLDKGNLSIFKSSNMNDEYTEFNTMYNDVPYLYFEDNNFDAEDILHNFLNDVVDNEVNFPVESTGNGNSFDVVTAKSFSSGDIETSNKPRKFVPFDVINPEYDTEELVDYFDYDYGLTTETKGTIEEFHGAKSPPKQVDLATENEADPWNKQMHLFPIHNIDANKNIFRNTTSEIFKTVDSLQKQNSSNPATAEENVNNFINIMQKASKNRHYQNQFQQIMLPNIFNTIESDDTLSFQKEDTNFYQDYNIDLNTVYQEINIILPNP

GTTPGIVTNSYKEIKKIVVPINVFTDGFAQIEGPSKEPVTFILLGTETTPGVVTEPNGQINIIYIPQILKNEGHMTLKIPTSTSKTNSMNQNSSPIESKLSIETPQKENYDTPYSSIIDQQLITQIDSVSGNQNQIQQVLQSNISHTILPDRTLSFREDNTISVQDVDKHFNTEIRKINILLPNP

GTTPGIVTNDYNEIKEIIVPNNIFANGFAQLKGPSGEPVTFILLGTETTPGVNGGEKDRSKSKPRNQIQQVMISLESDSVPKSTEEITKKQTIGTVIGPNNNIIYLVIQGY

GTTPGAVLDKNGEIKTIIVPENLELNQPVSINGPQNHPIIIVRKSSVNSPNIVTDSDNNISKIILPYSIAFKKSTPQELLMKSTTNSEARNPKRDFVNIIQKGGDNKNAPINQIQQIMISKDTQVHTRTTKPVVQSTMSEIDGDSEYQSTLPITLGSDQNSTESLTVPIGPLSSLFFNDSLKSLEIPFSIKHTGNISTSLNEVTVEPVFLFNPSYEINDTTTNTPFTVFVHSVPENHSSVESFTIPVEEASWMVSTNPGHFNLSLSNQISLESFTIPVGPLPLELSNTSSSSSQPIFTITNKGNDSFSNNEITAEPVLIVPTGEASNETKSQSSFTIPVHSMPTDSEVSFTIPRHEVFETVSRTPKSETFFEIGNQETESLPRKNNETVQSMSSLQIYLNSMNRKPEDNISIPNQFQINQFKFEIPTQPATNTTANKPSQFTGISFETDAKGTGLSLQKEENTKSFTVPKSFIWDKYFIKSLQPSNTTMEGGKGSYFIIPNNGSTFNSFNIWPTGQSKSKKDSQLLYVVPIEPIYVHDYPDSELTSKRKVESKKIKIDFLPKSSEKTNKYTFTVQVLPESGQKTTETTDFYQNFANQFLTMKDKAFQFPIELEGRRKSDLTKYLMKILFPRDRETSLFNTDVFMQKLELLNQFSSELITENLPIDLFDIFQEEHASNSLTLPIKLVDSILEKERELERHLPTFETGDIDFVPLNKEKS

LLSIFLGKKPHHSRI---PRIDTEDLDVSEREMELRRRLPASERRYFDLLPLKKKKY

LLSFFLEREPHHDIMSRMPRIDSEDLDIFEREMKLRRLLPASEIRDFDLLPLNKKKS

LLSFFLEREPHHDIMSRTPRIDSEDLEIFEREMELRRLE-SNLGYTYRGREKKDSSN

AAAINLDRQLPISNSKKIYKPKHQNHHNSINIKQTGNKGNQIQQVILPGGLKNDITSN

QLNFRETKYPVTDSITQRGISTKEKVKNGKIQTVKIPKHAIIRQTETTQSISVAMHSQEINKMQIIGTVTGPNSNIIYLVIQGY

GTTPGAVLDKNGEIKTIIVPENLELNQPVSINGPQNHPIIIVRKSSVTSPNIVTDSDNNIYKIILPYSIVFQ

NSTPEEPSRKSTTISVAHTHPAKNHLNIVQKNGREKDRSKTFEGRDFDLLPLNKKKS

LLSFFLEREPHHDIMSRTSRIDSEDLDFFEREIELRRRLPASERRYFDLLPLKKKKY

LLSFFLEREPHHDIMSRMPRIDSEDQEIFEREMELRRRLPASERRDFDLLPLKKKKS

LLSFFLEREPRHDIMSRMPRIDSKDLDIFEREMELRRLLPASERRDFDLLPLNKKKS

LLSFFLERETHHDLMSRTPRIDFEDLDFFEGEYKLGKRLPTSEARDFDILPLNKERS

LLSIFLERKPHDSIMSLIPHTFAKKEFLRFPRRSDSIMYRLFLDTIQNSREHDSLPSSHSIISLENSIFDNVHKHKHHILTEPNSHHHYVSHNKQNHLKAPIRKLQLEPSVRQKHFDPSIKETHLEPPIEHLHLKPSSKLENFDPSIKRTRLEPPSLLPETNSFSSTPKNSFLSNFVPMDEAERHGLLPSNVHQSRIFDSFSDKIRNFRKHEHFRPTQSINPTKSRMFTAEKPKHHRLR

EPSPYQRLTSATKYTHLERPNKRTHLEKPIRHVHLEPPSRLMHLEASIQSIDPTNSGMPNIYEKSKKHLLR

EPSPFQRLTSSTKHAHLEQPNKRMHLEKPSRHVHLEPPSELMHLEPSIQSIDPTNSGMSNI

EPSPFQRLASSTKHAHLGQPNKRTHLEKPSRYVHLEPPSELMHLEPLIQSIYPTNSRMSNIYEKPKKHLIR

EPSPYQRLASSTKHAHLEQPNKRKHLGTSSKRIHLELPNLSPEFTSFSPPTKFSYFSKSIPAIEAERRD

LLPPNRLQSTMFDLFSDKTQNYKEHELFPPTQFTNPTKSRIFNTFEMSQAHLITKPYPYHRFVSSTKRTHLKQPIKRTYLKKSIRDAHLEPPSERTHLEPSFKRTHLELPNLSPEFSSFSKFTKFSFPSKSISTIEAERRGLMPPNFHQSTIFDTFSDRVQNFKEHEHFPSMQSINPTKSRIFNMFERPKHHLLRES

FNYQNPATRMKQTHLQPPTFSIEDIPAFPHSERFDLIPPDIESTMSRLISKRNENHKLHSLPTMESIYPSESLMFSIFHKPKHHTLKKPTPQHLAKPEKRKHLKPPFILSESFPSFPELTSTRNTFHLNPVL

EIDTERDFLLPPIRDESLSSLKSFMTRNYENLDVFSPKQTPNSKESLFFKLFPHLKHHRLRES

AHHQHFVLSIDSAPTIEKWHMKPRTLLHEVFPIENMKFHETRHDEDFRRFFLHKIMDLTLDRGDHYMPKLFKILIESKPPEPEVLDFETLVPRVSSVFLAKKADYLSLGHNEVFVQLLLELLVTSLELIKAMDRSCMKSDFKILDKEIYTIGRDGDAGGAPLKLFLRRDIRRKFNAMITFCKVDSFISNPTTQSTSPNDDNPSEYLVPQAWRRRRLSSVTNLSTPIVFGHSKERNVQFQFTDLSSGIAKWKANYDLNENSSKRKLQRRGSTFFDDTFEDDEEFTEFMKYEDGTEEEINLSEEVTLFSKERNTSEEITIFTKERMRRPPSFNKQPRIEEEPKKDSWIVYLNAEAQSAPIEPLTSRLSKSFNTHRTSSVVVKQRNYYMTYPKLSQDKNLNSYKSTSELLKALKSYKSKNYRTVCEIFPKGTESLIDSPTENRNIVSL

Figure S3. Aggregate spidroin (AgSp1, AgSp2, AgSp-like) sequences with glycosylated and phosphorylated positions highlighted. Repeats units are aligned when possible.

**Figure S4**

Lh3tf006652g5u -----------------------------------------KMEV--SCLG-LISVLIVA

Lh3tf006652g2u ----------------LLEVPFLYQFLLQFHLQYFPIHKPYLPILSLFPVGLITFVFAVV

Lh3tf006652g10u --------------------------------------EPHRHHH-------VHHHHHGH

Lh3tf006652g4u EHFQRHWQPCQSSLCHSLDLVFLYL---QHLL---N-NYRSKIKMAVFNAVLVVLLLAVA

Lh3tf006652g3u ---------------------VLYF---SRSL---P-QDPSKMAV--SKAT-LSAILIIV

Lh3tf006652g1u --------------------------------------HPN-----------LGGNFNVD

Lh3tf006652g6u ------------------------------------------------------------

Lh3tf006652g5u AISGIESRPDPTVHHY---------RYKITSNEHTYGGGNG-GNVVN-------------

Lh3tf006652g2u FLDGIESRPQPAVHHY---------RYKIEDGGGDAGGEGG-ADFGG--------NPGGA

Lh3tf006652g10u LPGGEIKIPGGSGKVEIPGAGG---EVKIPGGGGEVKIPGGGGEVKIPGGGEKITIPGAS

Lh3tf006652g4u FPNGIESRPDPRVHHY---------RYKITSGTTHVND--------G-------------

Lh3tf006652g3u IISGIESRPDPTIHHY---------RYKITSNEHMHGGGDG-GNVMH-------------

Lh3tf006652g1u LNGGGHGQPGGGLNVNMNGGGRPNVGGNLNLNGGLGGGVGGGLNVGLNGEG----NGGSA

Lh3tf006652g6u ------ETKEPDVH----------------------------------------------

Lh3tf006652g5u -------------------------------------E----GEDLTNLGANAGDTRKRG

Lh3tf006652g2u FFQYKTSS---------------------KADLGHHHT----SLPHTNLVVDTGVARPRG

Lh3tf006652g10u GGKVEI------PGGGGEVK--IP---GGGGEVTVHGG----------------------

Lh3tf006652g4u -------------------------------------H----DEDLTNYGANAGDTRKRG

Lh3tf006652g3u -------------------------------------E----GEDLTNLGANAGDTRKRG

Lh3tf006652g1u GLNVNLNANGGLPNVGGNLNVGLNGGVGGGLDVNVNGKGTGGVGGSAHFIRTEGVGNTGK

Lh3tf006652g6u VHKYKTGK---------------------DVDVKDVDV----KKE-NKGPELPKFKRLRG

Lh3tf006652g5u EDCNRGLIDKAANFAGNTLNGAANKLESTVSGA-------TNKHGLLNNVGRGLDQLFTN

Lh3tf006652g2u TDCNRGLIDKAVAFAGHTLNGLASKLESTVSGI-------APEHGLVNNVGKGLDDLFEY

Lh3tf006652g10u ------------------------------------------------------------

Lh3tf006652g4u QDCGRGLIDKAANAAGNALTGAASGLENQVSGA-------TNKHGLLNNIGKGLDTLFEN

Lh3tf006652g3u EDCNRGLIDKAANFAGNTLNGAANKLESTVSGA-------TNKHGLLNNVGRGLDQLFTN

Lh3tf006652g1u ANCKKGIINGAANAAHNVLNGAIHGLQGGISKLTKGGHLLGPIGGILNTAGKAADDILTN

Lh3tf006652g6u EDCNRGLIDKAARFAGHTLNDAASGLENLVSRI-------APEHGLVNNVGKGLDALFEY

Lh3tf006652g5u GISGIRKGLTDAACGLGNIANEAADALDTGNIAGGVKNIAHEFFGGLGNIGADVTGAFSN

Lh3tf006652g2u GIRGIKRGLEDTACGLGNIADEIAEGIDTGKVPQEVKSIAREFFGGLGNIGRDITSAFSQ

Lh3tf006652g10u ------------------------------------------------------------

Lh3tf006652g4u GISGIRKGLDDAACGLGNIANEAASALDKGDIAGGVKNIAHEFFG---------------

Lh3tf006652g3u GISGIRKGLTDAACGLGNIANEAADALDTGNIAGGVKNIAHEFFGGLGNIGADVTGAFSN

Lh3tf006652g1u KISGINNVINDAACGLGDMANDAAEGLDTGNIVGGVGNMGKHFLGGIGNMAKDVVSGVGH

Lh3tf006652g6u GIRGIKRGLDDAACGLGSIANAIASGIDEGDLTGGVKEVAHEFFGGLGNIGRDVASAFSN

Lh3tf006652g5u AAEGLASDVGRLG---

Lh3tf006652g2u AAISLASDVSNLG---

Lh3tf006652g10u ----------------

Lh3tf006652g4u ----------------

Lh3tf006652g3u AAEGLASDVGRLG---

Lh3tf006652g1u ELGDTATDVKKGLFKG

Lh3tf006652g6u AAESIASDFQNLG---

Figure S4. Alignment of *L. hesperus* Aggregate Silk Factor 2 (AgSF2) homologs with phosphorylated positions highlighted.

**Figure S5**

MSTRG.30132.3 MMPNPR--------------VRKYLSTMKFYCLVIIFVCILNTARCQIPMGIPMGRPMGG

Lh3tf015943g12s -TSSPFLLHFTTTRKKNIIIINMRLLLVLAFCCLL--VIAAG------------------

Lh2tf028959g1s -----LYTGFHLCFKLNLNMKNSLLCLVLFFCFGIIYSMKVV------------------

MSTRG.30132.3 PMGALTSCQLQRLKTVMKNYPGMIIPKCNPDGSFQKKQCRNDAPICFCVNSLGLRIPHTL

Lh3tf015943g12s ----QSACRIQRKLVQQSGDKSAFLPRCTKDGKYAQIQCRQ--GFCWCAKSDGTQLTKSQ

Lh2tf028959g1s ----TSGCLRIRKFNEEIDFPGEYTPDCKKNGYFQPKQCRESIQYCWCVDKHGKVTHEPV

MSTRG.30132.3 SR-GPVEC-----

Lh3tf015943g12s KG--KPDCSNQPY

Lh2tf028959g1s PASENLQCPDNE-

Figure S5. Alignment of *L. hesperus* and *P. tepidariorum* modified Thyroglobulin Type-1 domain containing proteins with phosphorylated positions highlighted.


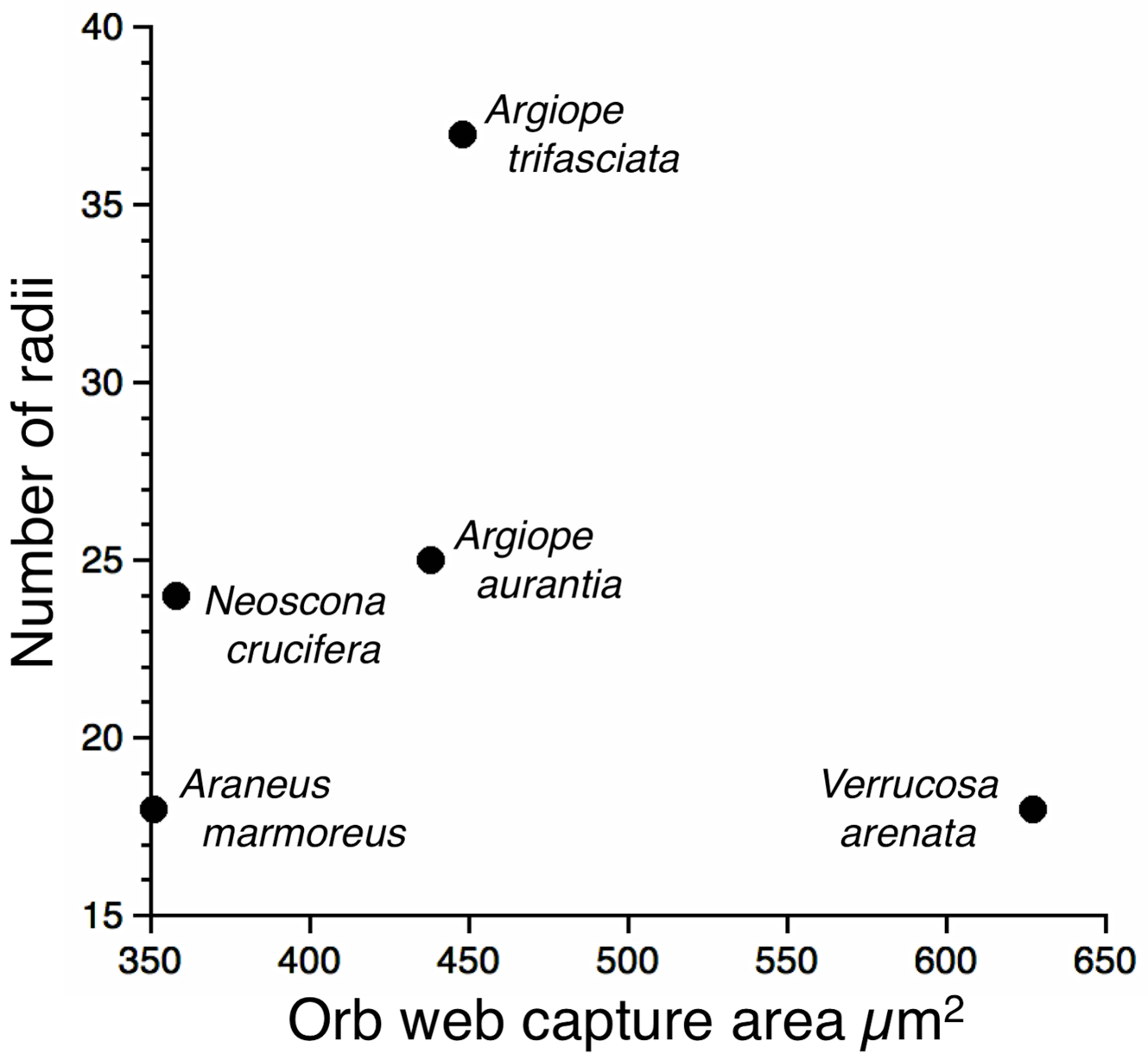


Figure S6. Comparison of the number of radii and prey capture areas of four orb weaving species whose adhesive properties have been characterized (Sensenig et al. 2010).
