# Supplementary material for: Protein Composition and Associated Material Properties of Cobweb Spiders’ Gumfoot Glue Droplets
Source: Integr Comp Biol. 2021 May 18;61(4):1459–80. doi: 10.1093/icb/icab086 (PMC8631074; doi:10.1093/icb/icab086)
Supplement: icab086_Supplementary_Data [file icab086_Supplementary_Data.zip › icb-2021-0105-File021.docx]

Please download the Supplementary Video using this link:

<https://wlu.box.com/s/32damgyu41rlsttxh4ol5g3cwavcb7ab>

Supplementary Videos. Movies of a *L. hesperus* droplet extended at 50% RH and of two droplets of the same individual *P. tepidariorum*, one extended at 40% and one at 60% RH. The probe tip in each movie has a width of 413 µm.
